# Comparative analysis of the RTFL peptide family on the control of plant organogenesis

**DOI:** 10.1007/s10265-015-0703-1

**Published:** 2015-02-21

**Authors:** Pin Guo, Asami Yoshimura, Naoko Ishikawa, Takahiro Yamaguchi, Youhao Guo, Hirokazu Tsukaya

**Affiliations:** 1 College of Life Science, Wuhan University, Wuhan, 430072 Hubei China; 2 Department of Biological Sciences, Graduate School of Science, University of Tokyo, Hongo, Tokyo, 113-0033 Japan; 3 Acel, Inc. SIC1 1201, 5-4-21 Nishihashimoto, Midori-ku, Sagamihara, Kanagawa Japan; 4 Present Address: Graduate School of Arts and Sciences, The University of Tokyo, 3-8-1 Komaba, Tokyo, 153-8902 Japan

**Keywords:** Evolution, Motif patterns, Organogenesis, Plant peptides, RTFL/DVL family

## Abstract

Plant peptides play important roles in various aspects of plant growth and development. The RTFL/DVL family includes small peptides that are widely conserved among land plants. Overexpression of six RTFL genes in Arabidopsis was suggestive of their functions as negative regulators of cell proliferation and as positional cues along the longitudinal axis of the plant body . At this time, few reports are available on RTFL paralogs in other species and the evolutionary relationship of RTFL members among land plants remains unclear. In this study, we compared and analyzed whole amino acid sequences of 188 RTFL members from 22 species among land plants and identified 73 motifs. All RTFL members could be grouped into four clades, and each clade exhibited specific motif patterns, indicative of unique evolutionary traits in the RTFL family. In agreement with this hypothesis, we analyzed two RTFL members from *Oryza sativa* and Arabidopsis by overexpressing them in Arabidopsis, revealing similar phenotypes suggestive of a conserved function of the RTFL family between eudicots and monocots, as well as different phenotypes and unique functions.

**Electronic supplementary material:**

The online version of this article (doi:10.1007/s10265-015-0703-1) contains supplementary material, which is available to authorized users.

## Introduction

To date, numerous peptides have been identified from plant genomes based on biochemical and genetic studies (Farrokhi et al. 2008; Kastin 2013). Peptides are defined as short chains of amino acid monomers; precursors are rarely larger than 120 amino acids and are typically present at low physiological concentrations (Katsir et al. 2011). Structurally, plant peptides can be categorized into two classes; secretory peptides and non-secretory peptides, based on the presence of an N-terminal secretory signal sequence (Matsubayashi 2014). These plant peptides play important roles in many processes including defense responses, callus growth, meristem organization, root growth, leaf-shape regulation, and nodule development (Matsubayashi 2014; Matsubayashi and Sakagami 2006). The secretory peptides act as signaling molecules for intercellular communication. CLAVATA3 (CLV3), ROOT MERISTEM GROWTH FACTOR1 (RGF1), and S-Locus cysteine-rich protein (SCR) or S-locus protein 11 (SP11) are well-characterized examples of secretory peptides (Fletcher et al. 1999; Kachroo et al. 2001; Matsuzaki et al. 2010; Schopfer et al. 1999).

Non-secretory peptides also play roles in the wounding/innate immunity response, nodule development, root growth, and leaf-shape regulation (Matsubayashi 2014; Matsubayashi and Sakagami 2006; Sakagami 2007). The non-secretory peptides are further divided into two types based on whether they act extracellularly or intracellularly (Matsubayashi 2014). Two well-characterized non-secretory peptides (Systemin and AtPep1) are known to act in the extracellular space; namely, Systemin (tomato systemin, TomSys), the first plant peptide discovered in wounded tomato (*Solanum lycopersicum*) leaves that can induce the production of proteinase inhibitors to defend against herbivore and pathogen attacks (Pearce et al. 1991); and *At*Pep1, the first known endogenous peptide elicitor that can be induced by wounding, methyl jasmonate, or ethylene, and activates innate immune responses such as the transcription of defensin, the production of H_2_O_2_, and the expression of its precursor gene (Huffaker et al. 2006). Although both TomSys and *At*pep1 are non-secretory peptides in structure and remain as precursors with a constitutively low expression level in the cytoplasm, the precursor proteins can be hydrolyzed into active peptides and released into the intercellular space upon cell wounding or pathogen invasion (Huffaker et al. 2006; Li et al. 2001; Meindl et al. 1998; Narváez-Vásquez and Ryan 2004; Narváez-Vásquez et al. 2005; Pearce et al. 1991; Scheer and Ryan 2002; Yamaguchi et al. 2006; Yamaguchi and Huffaker 2011). After binding to their receptors on the plasma membrane, wounding/defense information is transduced into intact cells to induce similar defense responses (Meindl et al. 1998; Scheer and Ryan 2002; Yamaguchi et al. 2006). Comparatively, EARLY NODULIN40 (ENOD40) and POLARIS (PLS) are considered typical non-secretory peptides that are synthesized and function intracellularly (Matsubayashi 2014). *ENOD40* is an early nodulin gene that is rapidly expressed during the invasion of rhizobia in the root pericycle and nodule primordium (Crespi et al. 1994; Kouchi and Hata 1993; Yang et al. 1993). Two short peptides (ENOD40A and ENOD40B) are directly translated from *ENOD40* mRNA (Röhrig et al. 2002). ENOD40 peptides strongly bind the cytosolic sucrose synthase (SuSy) enzyme (Chae et al. 2012; Röhrig et al. 2004) and are thought to activate sucrose cleavage and nodule development (Charon et al. 1999; Kumagai et al. 2006; Podkowinski et al. 2009; Takeda et al. 2005; Wan et al. 2007). PLS, a short open reading frame encoding a 36-amino-acid peptide, is required for correct auxin-cytokinin homeostasis to modulate root growth and leaf vascular patterning (Casson et al. 2002), and also negatively regulates ethylene responses to modulate cell division and expansion via the effects on cytoskeleton and auxin signaling (Chilley et al. 2006).

*ROTUNDIFOLIA4* (*ROT4*) was isolated through a gain-of-function genetic screen that resulted in a mutant called *rotundifolia4*-*1D* (*rot4*-*1D*) (Narita et al. 2004). This mutant exhibited shorter leaves with a reduced cell number mainly along the longitudinal axis in Arabidopsis. *ROT4* encodes a short peptide of 53 amino acids and negatively regulates cell proliferation in the longitudinal axis of organs, resulting in a phenotype of “small-round” rosette leaves (Narita et al. 2004; Wen et al. 2004). ROT4 is believed to be a non-mobile peptide synthesized without proteolytic processing since overexpressed ROT4-GFP and GFP-ROT4 localize on the plasma membrane and confer similar phenotypes (Ikeuchi et al. 2010; Narita et al. 2004). Overexpression of ROT4 under control of the heat shock promoter constructed using the Cre/Lox recombination system (Ikeuchi et al. 2010) suggests that ROT4 works cell-autonomously, which is indicative of its non-mobile characteristics. Wen et al. (2004) also identified a gene, *DEVIL1* (*DVL1*), from activation-tagged lines that develop horned fruit tips, which was later shown to be a paralog of *ROT4* in Arabidopsis. In total, 22 putative homologs of *ROT4* and *DVL1* were identified in the Arabidopsis genome, which were designated as the *ROT*-*FOUR*-*LIKE/DEVIL* (*RTFL/DVL*) family (Narita et al. 2004; Wen et al. 2004; Yamaguchi et al. 2013). RTFL/DVL peptides share a highly conserved domain of approximately 30 amino acids in the C-terminus, named the RTF domain (Narita et al. 2004). Overexpression of the RTF domain is sufficient to induce the *rot4*-*1D* phenotype (Ikeuchi et al. 2010). The remaining sequences of the RTFL/DVL family (especially in the N-terminal region) are poorly conserved and studied (Ikeuchi et al. 2010; Narita et al. 2004; Wen et al. 2004). Notably, RTFL/DVL members are highly variable in the length of their amino acid sequences (40–144 amino acids in Arabidopsis), suggestive of various roles or functions.

Our understanding of the biological function of the *RTFL/DVL* family is based on phenotypes observed in overexpression lines. Overexpression of at least six members of the *RTFL/DVL* family in Arabidopsis produces short-leaf phenotypes, which were similar to the *rot4*-*1D* mutant (Narita et al. 2004; Wen et al. 2004). Besides leaves, pleiotropic phenotypes in lateral organs are observed among overexpressors, such as shortened floral organs, protruding structures on the valves of fruits and at the base of pedicels, as well as trichomes (Ikeuchi et al. 2010). *ROT4/DVL16* suppresses polarized cell proliferation along the longitudinal axis, which mainly accounts for its effect on the shortened leaf phenotype (Ikeuchi et al. 2010) and the other lateral above-ground organs, which are considered to be leaf-derived organs (Golz and Hudson 2002; Valdivia et al. 2012). However, the loss-of-function lines provide little information on the biological function of the RTFL/DVL family. The reported insertional mutant of *RTFL4/DVL15* (Narita et al. 2004) and RNA interference constructs targeting *DVL1/RTFL18* and *DVL3/RTFL21* (Wen et al. 2004) did not produce any noticeable loss-of-function phenotype. Narita et al. (2004) also identified two insertional mutants within *Oryza* homologs, which were named *osrtfl1*-*1* and *osrtfl2*-*1*. Unfortunately, the mutants or mutant alleles did not show any discernable phenotypes, suggestive of a high level of genetic redundancy among the RTFL/DVL family.

The *RTFL/DVL* family is widely conserved among land plants (Floyd and Bowman 2007). However, the majority of studies have focused on RTFL*/DVL* members in Arabidopsis. The only report on a RTFL*/DVL* member in *Medicago truncatula* was published by Combier et al. (2008), which functioned as a negative factor to reduce nodulation. To explore the evolutionary processes and biological functions of this family, we compared the whole putative amino acids sequences of 188 RTFL/DVL members from liverworts to angiosperms and overexpressed one RTFL/DVL member from *Oryza sativa* in Arabidopsis. Comparative analysis was suggestive of an evolutionary trait of the RTFL/DVL family and revealed specific amino acids patterns (motif patterns) among species. The comparison could be used as background information for further study on the biological function of RTFL/DVL members in other species. Moreover, overexpression studies were suggestive of a conserved function of the RTFL/DVL family between monocots and eudicots in the control of plant organogenesis.

## Materials and methods

### Plant materials and growth conditions

The Arabidopsis accession Columbia (Col-0) was used as the wild type, and *p35S::ROT4* reported in Narita et al. (2004) was used as a reference line in this report. *Oryza sativa* L. cv. Taichung 65 (T65), a kind gift from Prof. Hiroyuki Hirano (The University of Tokyo), was used to isolate *OsRTFL3* (*Os01t0972300*). Transgenic lines of *p35S::OsRTFL3* were generated as described below. One single T-DNA insertion line (homozygous) was used for genetic and cytological analysis. Three independent heterozygous lines of *OsRTFL3 o/x* were used to confirm the root defects, as shown in Supplementary Fig. 2.

All Arabidopsis plants were grown on rock wool or Murashige and Skoog (MS) medium (Gamborg et al. 1976) at 22 °C under continuous light. T65 plants were grown in the container of clay loam soil under the same conditions. Young seedlings of Arabidopsis and T65 were collected 10 days after germination for reverse transcription polymerase chain reaction (RT-PCR) analysis. Arabidopsis used for root growth studies were cultured and grown on MS medium in the vertical direction.

### Vector construction and transformation

Total RNA was isolated from 10-day-old seedlings of T65 using the RNeasy Mini Kit (Qiagen, Valencia, CA, USA). The SuperScript one-step RT-PCR kit (Invitrogen, Carlsbad, CA, USA) was used for RT-PCR according to the manufacturer’s protocol. The amplification conditions of *OsRTFL3* using RT-PCR was one cycle at 95 °C for 2 min, followed by 35 cycles at 94 °C for 30 s, 55 °C for 30 s, and 72 °C for 1 min (2720 Thermal Cycler; Applied Biosystems, Foster City, CA, USA). The following pair of primers was used for amplification: OsRTFL3-Fw: 5′-CACCATGGAGGACGAGAGGTGGAAGC-3′ and OsRTFL3-Rev: 5′-CTAGTAGTCTCGCCAGCAGACGAG′. The RT-PCR product was cloned into the pENTRD-TOPO vector (Invitrogen) and then introduced into PH35G, a binary vector containing a Gateway cassette (Invitrogen) in the sense orientation under a CaMV 35S promoter (Narita et al. 2004).

The construct was introduced into wild-type Arabidopsis using *Agrobacterium*-mediated transformation with the simplified floral dip method (Clough and Bent 1998). Transgenic plants were selected on MS medium containing 2 mg mL^−1^ Gellan Gum (Wako, Osaka, Japan) and 20 μg mL^−1^ Hygromycin B (Aventis Pharma Ltd., Tokyo, Japan).

### Genomic PCR

Seven-day-old plants of wild type, OsRTFL3 o/x, and OsRTFL3 o/x-1–4 were used for genomic DNA isolation using the DNeasy Plant Mini Kit (Qiagen, Valencia, CA, USA). For the amplification of *OsRTFL3*, the following primers were used: OsRTFL3-F, 5′-ACTCGTCCGATTTCAACAGC-3′ and OsRTFL3-R, 5′-GGCGGACGATGTAGAACCT-3′. The amplification conditions using genomic DNA as template was one cycle at 95 °C for 2 min, followed by 20 cycles of touchdown PCR [94 °C for 30 s, 57 °C for 30 s (the temperature was reduced by 0.4 °C per cycle), and 72 °C for 90 s] and then 20 cycles of non-touchdown PCR (94 °C for 30 s, 55 °C for 30 s, and 72 °C for 90 s), with a final 72 °C for 7 min.

### Semiquantitative RT-PCR

For the amplification of *ROT4* and *OsRTFL3*, the following primers were used: OsRTFL3-F, 5′-GATTTCAACAGCAGCAACGC-3′; OsRTFL3-R, 5′-CGAATTGTTGCTCTGCTGCT-3′; ROT4-F: 5′-AGGAGAATGGCACGTGTGAG-3′; and ROT4-R: 5′-CAAGAGTCTTTGCGGTCGTG-3′. ACTIN2 (ACT2) was used as a control to detect constitutive expression. Primers for amplification of *ACT2* were as follows: ACT2-F, 5′-GAAATCACAGCACTTGCACC-3′ and ACT2-R, 5′-AAGCCTTTGATCTTGAGAGC-3′. The amplification conditions by RT-CPR was one cycle at 95 °C for 2 min, followed by 35 cycles at 94 °C for 30 s, 57 °C for 30 s, and 72 °C for 1 min.

### Comparative and phylogenetic analysis

The database used for searching sequences of RTFL members are listed in Table 1. To select RTFL members, HMMER was used to search the Pfam database and BLASTP was used to search SALAD, Pytozome v8.0, NCBI, and Sol genomics network (Table 1). Whole-sequence comparison of RTFLs was obtained from the Surveyed Conserved Motif Alignment Diagram and the Associating Dendrogram (SALAD) database (Mihara et al. 2009). SALAD is commonly used for genome-wide comparative analysis of annotated protein sequences in plants. The evolutionarily conserved short amino acid sequences in homologous proteins were identified as motifs using MEME software (http://meme.sdsc.edu/meme/intro.html) in SALAD. The motif significance is reported as the E-value upon MEME analysis, and the motif number in SALAD depends on this E-value. However, this numbering system sometimes results in dispersive motif numbers among the paralogs. In order to keep the motif numbers consecutive in the text, six motif numbers (Motif 5, 11, 12, 14, 16, 24) were slightly modified and re-numbered by considering a generality in distribution of each motif among various plant taxa (see the modification in Table S2). Annotations with similar motif patterns were grouped into the same clades according to the value of approximately unbiased (AU) or bootstrap probability (BP) (Shimodaira 2002, 2004). Clustering was calculated using pvclust in R software (http://www.r-project.org/).

**Table 1:** 188 RTFL members used in the comparative analysis

Species	Peptide abbreviation/Nomination	Database/Supple	Number of paralogs
Liverwort			
*Marchantia polymorpha*	MARPO	Prof. John L. Bowman	1
Moss			
*Physcomitrella patens*	PHYPA	SALAD^a^	2
Gymnosperm			
*Picea sitchensis*	PICSI	Pfam^b^	2
Monocotyledons			
*Oryza sativa*	From RAP-DB^c^	SALAD	20
*Brachypodium distachyon*	BRADI	SALAD	5
*Sorghum bicolor*	SORBI	SALAD	4
*Zea mays*	GRMZM	SALAD	10
*Hordeum vulgare* var*. distichum*	HORVD	Pfam	3
Eudicotyledons			
*Ricinus communis*	RICCO	Pfam	8
*Carica papaya*	CARPA	SALAD	2
*Glycine max*	GLYMA	SALAD	40
*Medicago truncatula*	MEDTR	SALAD	8
*Populus trichocarpa*	POPTR	SALAD	14
*Vitis vinifera*	VITVI	SALAD	2
*Fragaria vesca*	FRAVE	SALAD	3
*Arabidopsis thaliana*	RTFL1-23/ROT4	SALAD	24
*Arabidopsis lyrata* subsp. *lyrata*	ARALL	Pfam	17
*Thellungiella halophila*	THHALV	Pytozome v8.0^d^	7
*Thellungiella parvula*	THEPA	NCBI^e^	5
*Cleome spinosa*	ROSI	Pfam	2
*Solanum lycopersicum*	SOLYC	Sol genomics network^f^	3
*Aquilegia caerulea*	AQUCA	Pytozome v8.0	6
Total			188

^a^ http://salad.dna.affrc.go.jp/salad/en/

^b^ http://pfam.xfam.org

^c^ http://rapdb.dna.affrc.go.jp

^d^ http://www.phytozome.net

^e^ http://www.ncbi.nlm.nih.gov

^f^ http://solgenomics.net

Phylogenetic relationships between RTFLs in *O. sativa* and Arabidopsis were evaluated using MEGA version 6 (Tamura et al. 2013). A total of 43 RTF sequences of *O. sativa* and Arabidopsis were used for the analysis. Molecular Phylogenetic analysis was inferred using the maximum likelihood method based on the JTT matrix-based model (Jones et al. 1992). The bootstrap consensus tree inferred from 1000 replicates (Felsenstein 1985) was used to represent the evolutionary history of the taxa analyzed. Initial tree(s) for the heuristic search were obtained by applying the Neighbor-Joining method to a matrix of pairwise distances estimated using a JTT model.

### Anatomical analysis

The first leaves of 25-day-old plants were collected and cleaned with a chloral hydrate solution (4 g mL^−1^ chloral hydrate and 0.4 g mL^−1^ glycerol) as described by Tsuge et al. (1996). Palisade cells were observed using the Leica upright materials microscope (DM2700 M; Leica, Wetzlar, Germany). To record root growth, 3 days after germination a total of 30 Arabidopsis individuals of the wild type, *ROT4 o/x*, and *OsRTFL3 o/x* were scanned every 2 days. Individual cell sizes and root lengths were measured as described by Narita et al. (2004) using the ImageJ 1.48 program (National Institutes of Health; http://imagej.nih.gov/ij/).

## Results

### Comparative analysis of the RTFL family

**Fig. 1:**
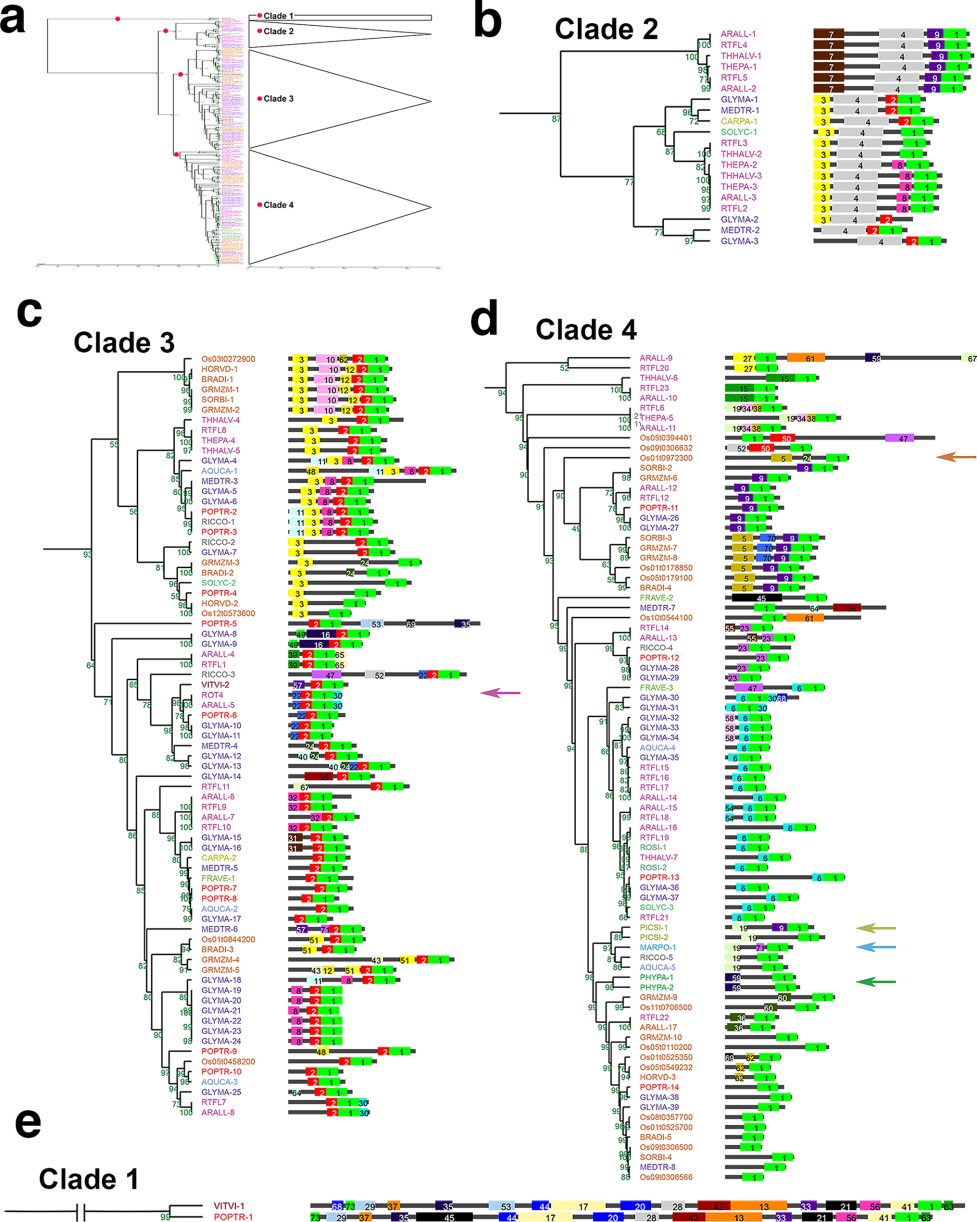
Comparative analysis of the 180 RTFL family among land plants. The whole tree (**a**) was generated using SALAD (Mihara et al. 2009) and divided into four clades (**b**–**e**) based on the bootstrap values, and was approximately unbiased. Only bootstrap values were shown beside the branches (in *green*). Full amino acid sequences of RTFLs were used for analysis. The complete tree is shown in Fig. S1. **c** *Arrow* indicates ROT4. **d** *Arrows* (from *top* to *bottom*) indicate RTFLs of *Oryza sativa* (OsRTFL3), *Picea sitchensis*, *Marchantia polymorpha*, and *Physcomitrella patens*, respectively. RTFLs of species from one family were marked with the *same color* in the figure. Motifs were numbered in *different colors* in the *boxes*. *Green* numbers around the branches indicated the bootstrap values

The RTFL family is widely conserved among land plants and shares no sequence similarities with identified proteins or well-characterized motifs (Narita et al. 2004; Wen et al. 2004). Therefore, we investigated the biological functions of RTFL orthologs to increase our understanding of their effects on the control of plant organogenesis. SALAD is a motif-based database for plant comparative proteomics. This program can be used to predict biological function based on the hypothesis that proteins with similar motifs have similar biochemical properties and thus related biological functions (Mihara et al. 2009). Consequently, we collected 188 RTFLs among 22 species with full-length amino acid sequences for comparative analysis using SALAD (Table 1, see the whole amino acid sequences of 188 RTFLs in Supplemental Table S1). A total of 8 RTFLs were excluded by SALAD due to the low similarity calculated using the MEME software (Bailey et al. 2009), and the remaining 180 RTFLs were shown in the comparative analysis (Fig. 1, see the complete tree in Fig. S1). These sequences cover a wide range of land plant lineages, including liverworts, moss, gymnosperms, and angiosperms.

A total of 73 motifs were identified using the MEME suite (Bailey et al. 2009) among 180 RTFLs (see all motif sequences in Supplemental Table S2; see the nomination rules of motifs in the “Materials and Methods”). The RTF domain, which was used for blasting RTFL members, was presented as Motif 1 among all RTFLs (Figs. 1, S1). The N-terminal region of the RTFL family is less conserved among RTFL members, and no predictable signal peptides have been identified (Narita et al. 2004; Wen et al. 2004). This was confirmed by our results since the remaining 72 variable motifs (Motifs 2–73) were mostly identified around the N-terminus without any signal motifs (Figs. 1, S1).

The 180 RTFLs were grouped into four clades (Clades 1–4; Fig. 1) based on the motif patterns. Conserved motifs could be found in an interspecific manner. Excluding Motif 1 that defined the RTFL family, Motifs 2–12 were found in various species from liverworts to angiosperms (Figs. 1, S1). In Clade 4, Motifs 9, 19, 59 and 71 were found in *Marchantia polymorpha* (liverwort), *Physcomitrella patens* (moss), and *Picea sitchensis* (gymnosperm) (Figs. 1d, blue, green and yellow-green arrows, S1, respectively). Motifs 2 and 3 were found among RTFLs of eudicots and monocots in Clade 2 and Clade 3 (Figs. 1b, c, S1). In addition, Motifs 2–4 and 7–9 were found among species in Clade 2, which consisted of only eudicots (Figs. 1b, S1). Clade 1 consisted of two long RTFLs with diverse motifs (Figs. 1e, S1). Conserved motifs could also be found within specific families. Two types of motif combinations (Motifs 3, 4 and 8; Motifs 4, 7, and 9) were specific in Brassicaceae, and were also observed in *Arabidopsis thaliana*, *A. lyrata* subsp. *lyrata*, *Thellungiella parvula*, and *T. halophila* (Figs. 1b, S1). Motifs 2–4 and 8 were conserved among RTFLs in *Glycine max* and *M. truncatula* of Leguminosae (Figs. 1b, c, S1). Motifs 2, 3, and 10 were conserved among all species of Gramineae, which we compared to *O. sativa*, *Brachypodium distachyon*, *Sorghum bicolor*, *Zea mays*, and *Hordeum vulgare* var. *distichum* (Figs. 1c, S1). According to the motif patterns, RTFL members in Arabidopsis could be divided into five subgroups (Table 2); Subgroup 1 contained Motifs 1, 3 and 4, which were shared by RTFL 2 and 3 in Clade 2 (Figs. 1b, S1); RTFL4 and 5 in Clade 2 (Figs. 1b, S1) were grouped into Subgroup 2, both of which contained Motif 1, 4, 7 and 9; Subgroup 3 included ROT4, RTFL1 and RTFL 7–11 in Clade 3 (Figs. 1c, S1), with a pattern of Motifs 1 and 2 in common; RTFL15–19 and 21 in Clade 4 (Figs. 1d, S1) were grouped into Subgroup 4, with Motifs 1 and 6 in common; the remaining RTFLs (RTFL 6, 12, 13, 14, 20, 22 and 23) in Clade 4 (Figs. 1d, S1) showed diverse motif patterns with only Motif 1/functional RTF domain in common, and thus were grouped into Subgroup 5 (RTFL13 was excluded by SALAD in Figs. 1, S1, but was included in the same clade with RTFL14 when analyzed with lower amounts of RTFLs, unpublished data).

**Table 2:** Subgroups of 24 RTFL members in Arabidopsis

Motifs	1	2	3	4	6	7	9
RTFLs							
Subgroup 1 (Clade 2)							
RTFL2	◯		◯	◯			
RTFL3	◯		◯	◯			
Subgroup 2 (Clade 2)							
RTFL4	◯			◯		◯	◯
RTFL5	◯			◯		◯	◯
Subgroup 3 (Clade 3)							
ROT4	◯	◯					
RTFL1	◯	◯					
RTFL7	◯	◯					
RTFL8	◯	◯					
RTFL9	◯	◯					
RTFL10	◯	◯					
RTFL11	◯	◯					
Subgroup 4 (Clade 4)							
RTFL15	◯				◯		
RTFL16	◯				◯		
RTFL17	◯				◯		
RTFL18	◯				◯		
RTFL19	◯				◯		
RTFL21	◯				◯		
Subgroup 5 (Clade 4)							
RTFL6	◯						
RTFL12	◯						
RTFL13	◯						
RTFL14	◯						
RTFL20	◯						
RTFL22	◯						
RTFL23	◯						

Circles represent the related motifs found in the corresponding RTFL members. Only the motifs shared by all members in a subgroup were shown in the table

### Phylogenetic analysis of RTFL members in Arabidopsis and* O. sativa*

**Fig. 2:**
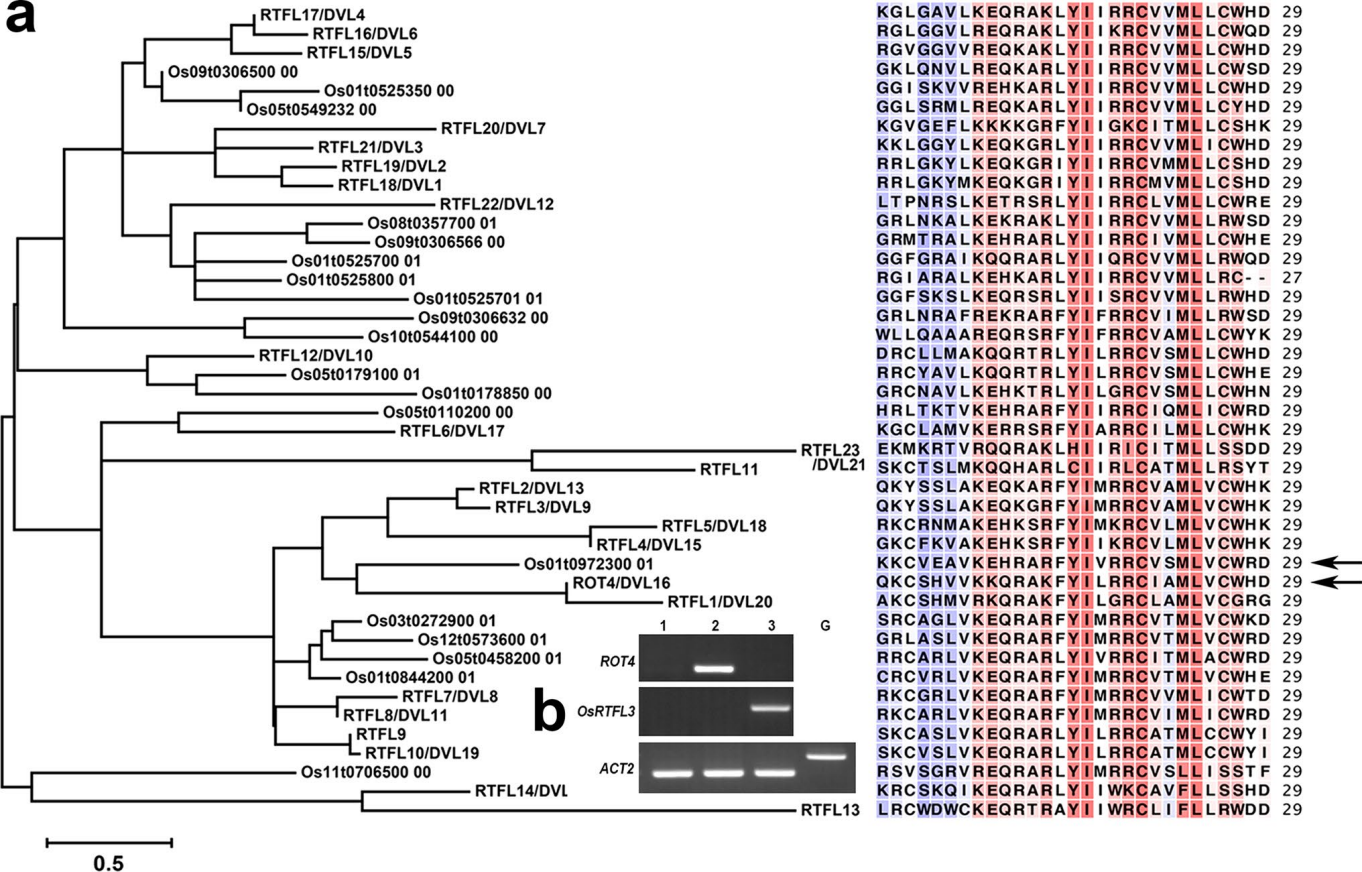
Molecular phylogenetic analysis of RTFL members. **a** Phylogenetic analysis and alignment of 43 RTFL members from *Oryza sativa* and Arabidopsis. RTF domains, which show homology at the C-terminus of the RTFL family, were aligned using the ClustalW software and constructed using MEGA 6.0 using the maximum-likelihood method. *Arrows* indicate Os01t0972300_01 (*upper*) and ROT4 (*below*). **b** RT-PCR analysis of *ROT4* and *OsRTFL3* mRNA accumulation. Total RNAs of mature 10-day-old whole plants were used. *Lane 1*, wild type; *lane 2*, *ROT4 o/x*; *lane 3*, *OsRTFL3 o/x*. *ACT2* was used as an internal control. *Lane G* shows a negative control using genomic DNA of 10-day-old wild-type plants

Based on the above comparative analysis, we examined RTFL diversity between Arabidopsis (an eudicot) and *O. sativa* (a monocot), both of which are common model plants that have been fully sequenced (The Arabidopsis Genome Initiative 2000; mads Genome Sequencing Project 2005). A total of 90 % of Arabidopsis genes are believed to have homologs in the rice genome (International Rice Genome Sequencing Project 2005), and here 20 RTFL orthologous members in *O. sativa* exhibited diverse motif patterns (Table 3; Figs. 1, S1). Therefore, we generated a phylogenetic tree of 43 RTFL members from Arabidopsis and *O. sativa* (Fig. 2a) based on the conserved RTF sequences (identified as Motif 1 in Figs. 1, S1) for two reasons: (a) RTF domain/Motif 1 of Arabidopsis is sufficient to induce the RTFL-overexpression phenotypes in leaves and fruits (Ikeuchi et al. 2010); (b) RTF domain/Motif 1 is the only sequence/motif conserved among all RTFLs in Arabidopsis and *O. sativa* (Figs. 1, S1). The short length of RTF domains resulted in weak bootstrap values (data not shown), but the general topological relationships were observed regardless of the analysis parameters. Os01t0972300 was phylogenetically clustered into the same clade with ROT4 (Fig. 2a; arrows) based on RTF sequences/Motif 1, although they were in different clades based on the comparative analysis of whole amino acids sequences/whole motif patterns (Fig. 1c, d, purple and orange arrows). *Os01t0972300* encodes 124 amino acids and was named *OsRTFL3* in this report, which follows the nomination of *OsRTFL1* and *OsRTFL2* in Narita et al. (2004).

**Table 3:** Species of RTFLs which exhibit similar motif patterns as ROT4, DVL1–5 and OsRTFL3

Species of RTFLs	Paralog numbers	Published RTFLs with similar motif patterns
*Vitis vinifera*	2	ROT4
*Ricinus communis*	6	ROT4
*Sorghum bicolor*	4	OsRTFL3
*Brachypodium distachyon*	5	OsRTFL3
*Fragaria vesca*	3	DVL1–5
*Aquilegia caerulea*	6	DVL1–5
*Solanum lycopersicum*	3	DVL1–5
*Cleome spinosa*	2	DVL1–5

### OsRTFL3 has similar functions as ROT4 in the development of above-ground organs

The long evolutionary history and conserved sequence of RTFL peptides are indicative of their essential functions in land plant evolution. To investigate whether the RTFL family is functionally conserved between eudicots and monocots, *OsRTFL3* was constructed under the 35S promoter of the *Cauliflower mosaic virus* (CaMV35S) and transformed into wild-type Arabidopsis (Col-0). We established five independent transgenic lines and selected one line for further study after confirming that all individuals showed fundamentally similar phenotypes. Transgenic plants overexpressing *ROT4* were used in Narita et al. (2004), with the coding sequence of *ROT4* constructed under CaMV35S. The high expression level of *ROT4* and *OsRTFL3* was confirmed using RT-PCR in the transgenic plants (Fig. 2b). No detectable amplification of *ROT4* was observed in the wild-type Arabidopsis under our PCR conditions, which could be explained by the low expression level. These two overexpressing lines were termed *ROT4 o/x* and *OsRTFL3 o/x* in the following content.

**Fig. 3:**
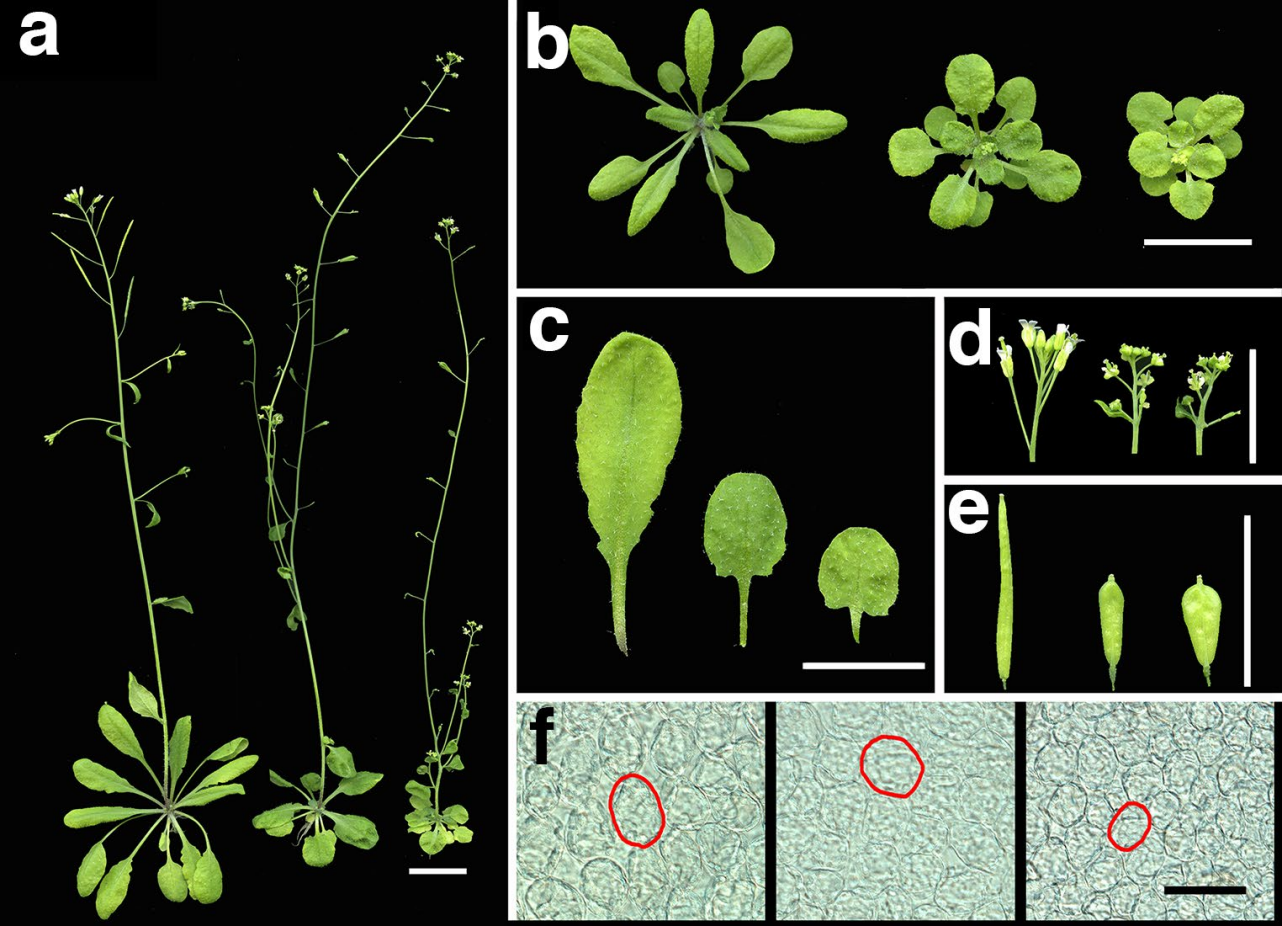
Phenotypes of *OsRTFL3 o/x*. **a** Thirty-five-day-old plants of wild-type Arabidopsis, *ROT4 o/x*, and *OsRTFL3 o/x* (from *left* to *right*). **b** Twenty-five-day-old rosette leaves of the wild type, *ROT4 o/x*, and *OsRTFL3 o/x* (from *left* to *right*). **c** The fifth rosette leaves of the wild type, *ROT4 o/x*, and *OsRTFL3 o/x* (from *left* to *right*). **d** Inflorescences of the wild type, *ROT4 o/x*, and *OsRTFL3 o/x* (from *left* to *right*) plants. **e** Fruits of 35-day-old wild type, *ROT4 o/x*, and *OsRTFL3 o/x* (from *left* to *right*). **f** Palisade cells in the middle portion of first foliage leaves of 25-day-old wild type, *ROT4 o/x*, and *OsRTFL3 o/x* (from *left* to *right*) plants. *Red circles* indicate a single palisade cell. *Scale bars* = 1 cm (**a**–**e**); 100 µm (**f**)

**Fig. 4:**
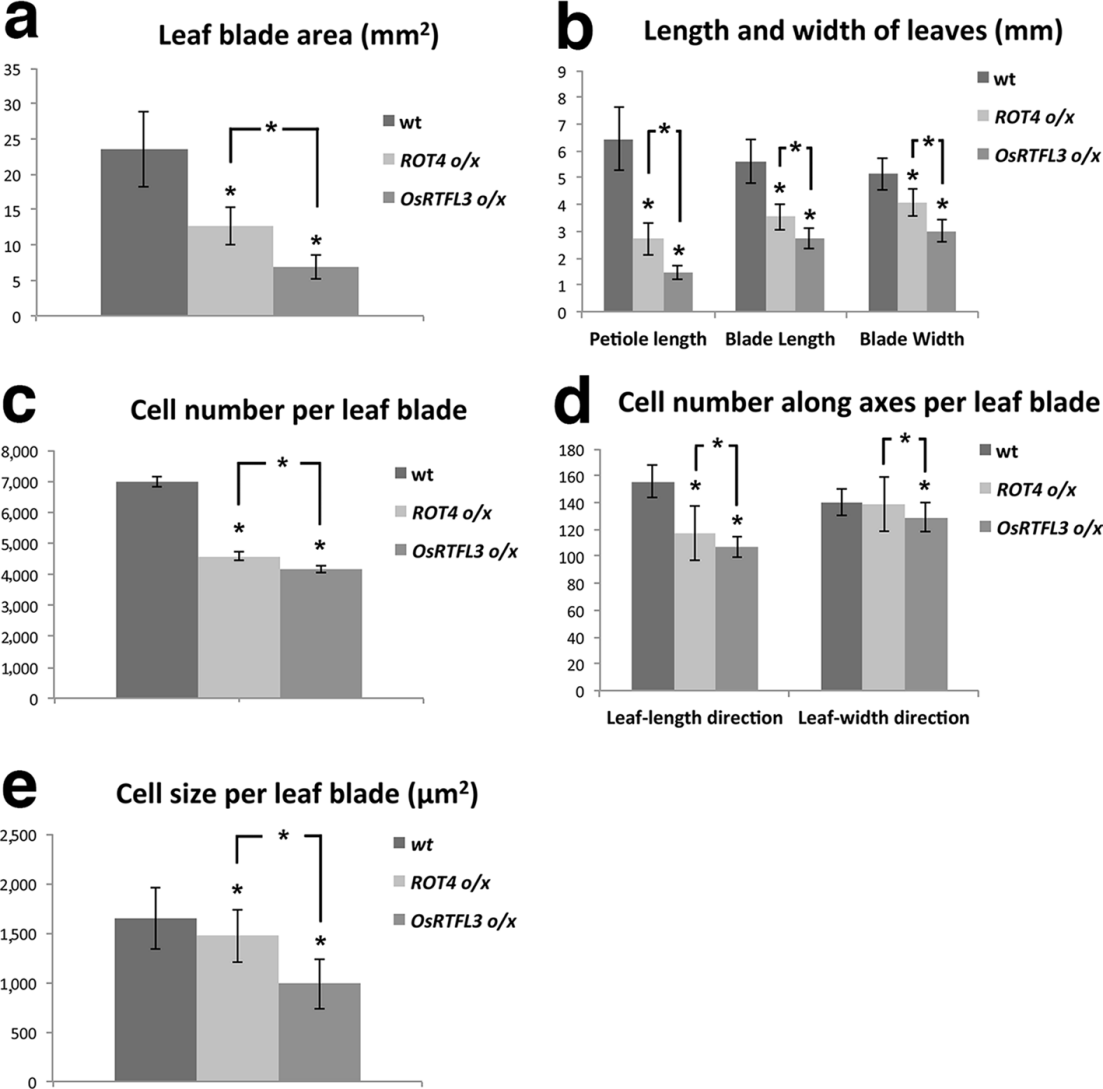
Measurement of leaf morphological characteristics in *ROT4 o/x* and *OsRTFL3 o/x* **a** The blade area of 25-day-old wild type (*left*), *ROT4 o/x* (*middle*), and *OsRTFL3 o/x* (*right*). *n* = 10. Values represent the mean ± SD. **b** Leaf petiole, blade length, and width of first leaves of 25-day-old wild type (*left*), *ROT4 o/x* (*middle*), and *OsRTFL3 o/x* (*right*) were measured using ImageJ 1.29 program *n* = 10. Values represent the mean ± SD. **c**, **d** Numbers of palisade cells of the first leaf blade of 25-old-day plants. **c** Numbers of palisade cells in the subepidermal layer per leaf blade; **d** Numbers of palisade cells in leaf-length and leaf-width directions. The columns: wild type (*left*), *ROT4 o/x* (*middle*), and *OsRTFL3 o/x* (*right*) *n* = 10. Values represent the mean ± SD. **e** Palisade cell size of the first leaf blade of 25-old-day plants. Cell area of 10 cells was measured for each line. Columns indicate palisade cell size of the wild type (*left*), *ROT4 o/x* (*middle*), and *OsRTFL3 o/x* (*right*) *n* = 10. Values represent the mean ± SD. *Asterisk* indicates significant differences calculated using Student’s *t* test (*P* < 0.001)

We next compared the morphology of wild-type plants, *ROT4 o/x*, and *OsRTFL3 o/x*. Both *ROT4 o/x* and *OsRTFL3 o/x* showed a pronounced reduction in organ size (Fig. 3a, b). The reduction in blade area, petiole length, blade length, and width of *OsRTFL3 o/x* was more significant when compared with wild type and *ROT4* *o/x* (Figs. 3c,4a, b; *P* < 0.001, paired student’s t test). *OsRTFL3 o/x* also showed a short-organ phenotype in inflorescences and fruits, similar to *ROT4 o/x* (Fig. 3d, e). In addition, fruits of *OsRTFL3 o/x* were wider than those of wild type and *ROT4 o/x* (Fig. 3e). Although filaments and stamens were much shorter in *OsRTFL3 o/x*, they could reach the stigma at later developmental stages. Therefore, *OsRTFL3 o/x* was fully fertile, as was *ROT4 o/x* (Narita et al. 2004). The above comparison of gross morphology demonstrated that phenotypes of *OsRTFL3 o/x* in shoots were similar to *ROT4 o/x,* but were quantitatively different.

Organ size is determined by both cell size and number (Tsukaya 2006). To examine whether the reduced leaf size of *ROT4 o/x* and *OsRTFL3 o/x* were induced by a decrease in cell number and/or size, the number and size of palisade cells in the first rosette leaves of wild-type plants, *ROT4 o/x*, and *OsRTFL3 o/x* were measured. The total number of palisade cells per leaf blade in both *ROT4 o/x* and *OsRTFL3 o/x* decreased significantly, with a more severe reduction in *OsRTFL3 o/x* (Fig. 4c). To confirm whether the decreased cell number was related to the effect of *ROT4 o/x* and *OsRTFL3 o/x* on leaf shape, the palisade cell numbers in both the leaf-length and leaf-width direction were counted. The results showed that the cell number of *OsRTFL3 o/x* and *ROT4 o/x* in the leaf-length direction decreased in a similar pattern as the decrease in total cell number in the subepidermal layer. However, cell numbers along the leaf-width direction in *OsRTFL3 o/x* significantly decreased compared with wild type and *ROT4 o/x* (Fig. 4d). Similarly, the size of palisade cells in both *ROT4 o/x* and *OsRTFL3 o/x* significantly decreased, with a more severe reduction in *OsRTFL3 o/x* (Fig. 4e). This pattern indicates that both *ROT4 o/x* and *OsRTFL3* control polar cell proliferation as well as cell expansion in the lateral organs, suggesting that *OsRTFL3* has a similar function as *ROT4* in the control of organogenesis when overexpressed in Arabidopsis. In addition, *OsRTFL3* showed a unique function in negatively regulating the cell number along the leaf-width axis when overexpressed, which was not observed in *ROT4 o/x* lines.

### OsRTFL3 o/x inhibited root growth

**Fig. 5:**
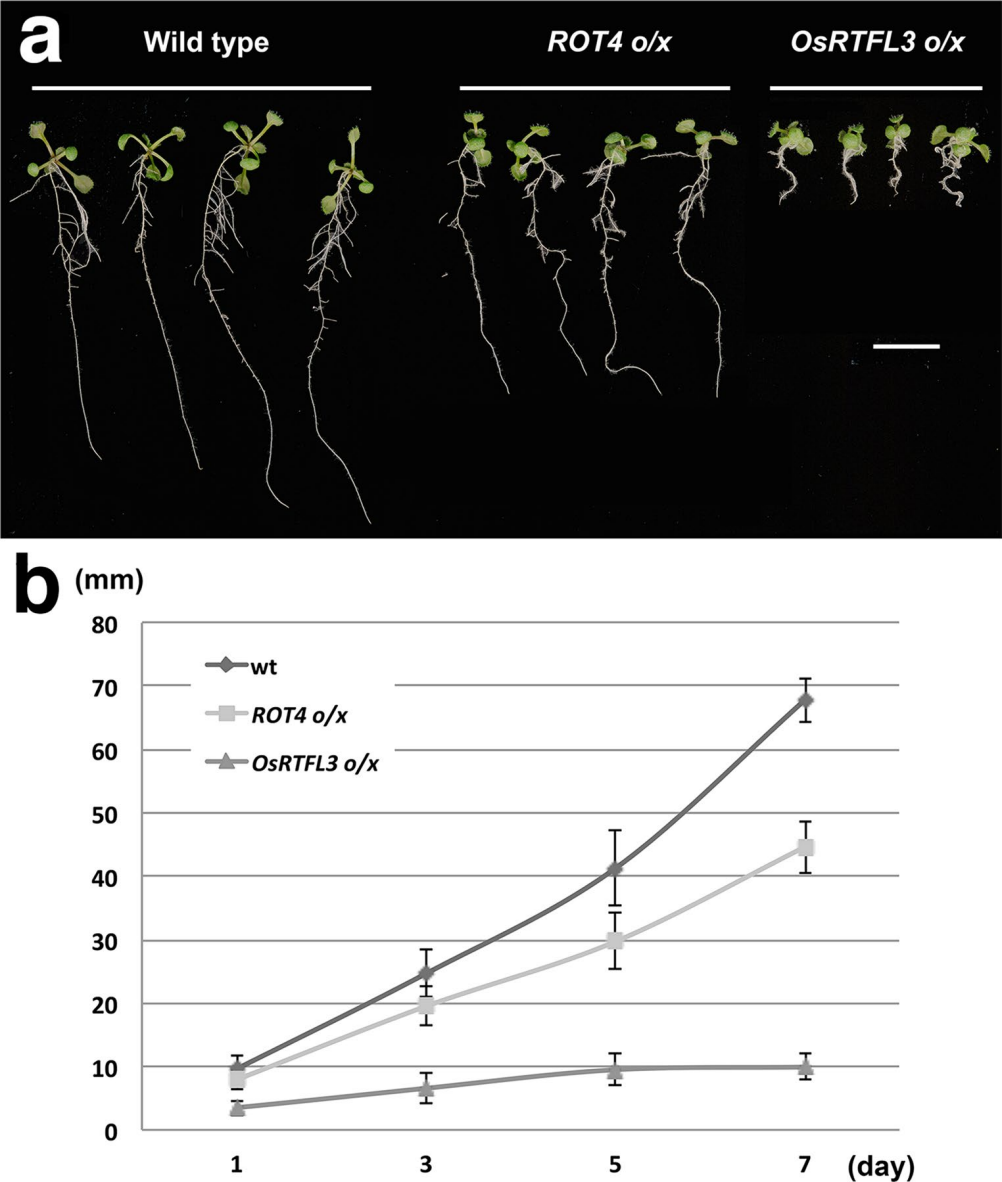
Root growth of 10-day-old wild type (*left*), *ROT4 o/x* (*middle*), and *OsRTFL3 o/x* (*right*) plants. **a** Root phenotype of 10-day-old plants. **b** Time-course analysis of root length *n* = 30, *Scale bars* = 1 cm

Although at least six RTFL members in Arabidopsis have been overexpressed and resulted in the dominant “round-leaf” phenotype, no significant differences were observed in morphological features of roots between wild type and RTFL overexpressors (Narita et al. 2004; Wen et al. 2004). However, according to our observations, both *ROT4 o/x* and *OsRTFL3 o/x* generated shorter primary roots, and *OsRTFL3 o/x* exhibited a more severe phenotype (Fig. 5a). The rate of root elongation decreased severely in *OsRTFL3 o/x* based on the time-course analysis, which was also observed in *ROT4 o/x*, but more mildly. (Fig. 5b). In addition, the roots of *OsRTFL3 o/x* almost stopped elongating around the fifth day after germination, while the elongation rate began to accelerate in the wild type and *ROT4 o/x*. Inhibition was observed in another three independent lines of *OsRTFL3 o/x*, and all *OsRTFL3 o/x* lines had a capability of generating lateral roots (Figs. 5a, S2). The developmental defects in root growth of both *ROT4 o/x* and *OsRTFL3 o/x* were inconsistent with the previous RTFL-overexpressing phenotypes observed in Arabidopsis. The phenotypes of *OsRTFL3 o/x* regarding the regulation of cell numbers along the leaf-width axis and root growth suggested that *OsRTFL3 o/x* may have unique functions in the control of organogenesis, in addition to the common functions as *ROT4*.

## Discussion

### Diversity of the N-terminal structure among RTFLs

All RTFLs share a highly conserved domain in the C-terminus (RTF domain) and diverse sequences in the N-terminus. Examining the diversity of the N-terminal structure is one application of comparative RTFL analysis.

Changes in motif architecture preferentially occur at the protein termini since they are more tolerant to domain insertions or deletions due to terminal flexibility (Bjorklund et al. 2005; Buljan and Bateman 2009; Weiner et al. 2006). This domain shaping may also occur in the ancestral sequence of the RTFLs. The domains/motifs of the RTFL family are quite short, ranging from 6 to 35 amino acids (examples are shown in Table S2). Modulation of the RTFL motifs may not incur large alternations at the gene level compared with other “normal-size” proteins with an average length of 120 (Buljan and Bateman 2009). Thus, the RTFL peptide family may be more tolerant to domain changes due to the small sequence sizes, which may explain the diverse structure of the N-terminus in the RTFL family.

### Motif patterns of RTFL members

This is the first study to perform a comparative analysis of the RTFL family. In total, 180 RTFL members from 22 species were grouped into four clades (Clades 1–4 from top to bottom of the tree; see Fig. 1b–e). RTFLs of *Marchantia polymorpha*, *Physcomitrella patens*, and *Picea sitchensis*, which share an early evolutionary position among land plants, were clustered into Clade 4 (Fig. 1d, green and yellow-green arrows), indicating that the motif patterns in Clade 4 may represent the ancestral structure of the RTFL family. Clade 3 (Fig. 1c) exhibited diverse motif patterns, and members in this clade were found in all flowering plants examined, including Arabidopsis (a dicot) and *Oryza sativa* (a monocot). This suggested that an evolutionary event occurred in the RTFL family during the initiation of flowering plants, and that the RTFL family may have gained additional motifs after divergence from its ancestors. The majority of RTFLs in Clade 3 contain Motif 2, indicating that the formation of Motif 2 was associated with the basic function of Motif 1 in the RTFL family among flowering plants (Fig. 1c). RTFL members in Clade 2 (Fig. 1b) shared uniform motif patterns and were observed only in eudicots. Excluding functional Motif 1, Motif 4 was also found among the RTFLs in Clade 2, suggestive of its specific role in the evolutionary process of eudicots (Fig. 1b). Meanwhile, all RTFLs of Gramineae (monocots) in Clade 3 contained motifs 2, 3, and 10, suggesting that these motifs play specific roles in monocot evolution (Fig. 1c). Based on the motif patterns, the RTFL family may have originated from early bryophytes and experienced an evolutionary event during the transition to flowering plants. The required new motifs, which were formed after the transition, may have different roles in the evolution of flowering plants and some specific families.

### Functions of motifs in the RTFL family

The primary purpose of the SALAD program is biological and biochemical prediction. Proteins with similar motifs/motif patterns are assumed to have related biological functions (Mihara et al. 2009). Wen et al. (2004) overexpressed DVL1/RTFL18, DVL2/RTFL19, DVL3/RTFL21, DVL4/RTFL17, and DVL5/RTFL15 in Arabidopsis, which exhibited similar phenotypes, suggestive of their similar biological functions. These DVLs/RTFLs were clustered into the same subgroup based on motif patterns (Table 2, Subgroup 4), which supported the accuracy of the SALAD program in the aspect of biological and biochemical prediction in our study. Thus, RTFL candidates clustered closely to the reported RTFL members (ROT4/DVL16, DVL1/RTFL18, DVL2/RTFL19, DVL3/RTFL21, DVL4/RTFL17, DVL5/RTFL15, and OsRTFL3) in the comparative analysis should be further explored; namely, VITVI–2 of *Vitis vinifera*, RICCO–3 of *Ricinus communis* (two motif patterns similar to ROT4, Fig. 1c), SORBI–2 and SORBI–3 of *Sorghum bicolor*, BRADI–4 of *Brachypodium distachyon* (grouped into the same clade with OsRTFL3, Fig. 1d), FRAVE–3 of *Fragaria vesca*, AQUCA–4 of *Aquilegia caerulea*, SOLYC–3 of *Solanum lycopersicum*, and ROSI–1 and ROSI–2 of *Cleome spinosa* (motif patterns similar to DVL1–DVL5, Fig. 1d). The biological function of the above 10 candidate members are thought to exhibit similar motif patterns as the reported RTFL members in Arabidopsis and *O. sativa*, which negatively regulate cell proliferation in the polar direction. Meanwhile, the numbers of RTFL paralogs in the above 10 candidate species are quite small, ranging from two (*V. vinifera*) to six (*R. communis*) (Tables 1, 3). Further genetic studies on the above 10 candidate members, especially the construction of loss-of-function mutants, may increase our understanding of the exact function of the RTFL family since our current knowledge is limited due to the high gene redundancy of RTFLs in both Arabidopsis and *O. sativa*. In addition, genome information on the above candidates can be found on the related web sites.

**Fig. 6:**
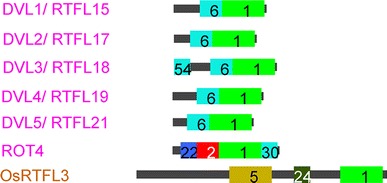
Motif patterns of DVL1–5, ROT4, and OsRTFL3 (Modified from Figs. 1, S1)

Wen et al. (2004) reported that a wide range of phenotypic variation, (besides the common “short-leaf” phenotype), especially in fruits, was observed among the transgenic lines overexpressing DVL1–DVL5. Ikeuchi et al. (2010) demonstrated that overexpression of the core functional RTF region of ROT4 (Motif 1 in our study) is sufficient to induce the fruit phenotype. Thus, the remaining non-conserved sequences or motifs are responsible for the phenotype variation in fruits. Indeed, we previously found that two truncation lines overexpressing the ROT4 functional RTF domain with a deletion of non-conserved sequences in the N-terminus and C-terminus were similar to full-length *ROT4 o/x,* exhibiting the “short-leaf” phenotype, but showed a variation in the fruit phenotype (Narita et al. 2004). Similarly, Ikeuchi et al. (2010) also observed variations in fruit shape, and the frequency of the protrusion of inflorescence stems varied among a series of truncations when *ROT4* was overexpressed (Ikeuchi et al. unpublished observation), supporting the above hypothesis. Hence, we examined *OsRTFL3* from rice and demonstrated that the “sub-phenotypes” of *OsRTFL3 o/x* differed in fruit shape, cell number, and root development, while the common “short-leaf” phenotype was observed. According to our motif alignment (Fig. 1), no common motifs exist among ROT4, DVL1–DVL5, or OsRTFL3, excluding the functional Motif 1 (Fig. 6). Since the Motif 1/RTF domain is functional in the RTFL family and induces dominant phenotypes, all RTFL overexpressors generate similar “short-leaf” phenotypes (Ikeuchi et al. 2010). However, the “sub-phenotypes” vary among samples, which suggests that the core RTF region (Motif 1) may be responsible for the “short-leaf” phenotype and that N-terminal motifs or the non-conserved sequences in RTFL members may perform specific functions contributing to the variable phenotypes. The possibility remains that such phenotypic variations can be caused by positional effects, namely, the fluctuating expression levels among transgenic plants. However, an examination of a series of comparisons among independent lines does not support this hypothesis.

### Perspectives on the study of the RTFL family

The high level of gene redundancy hinders studies on the RTFL family. Without analyzing loss-of-function mutants, we could not thoroughly examine RTFL function. Based on our results, species with a lower number of paralogs carrying similar motif patterns as the reported RTFLs could be used to establish and study the loss-of-function lines. In addition, we observed a lower level of *RTFL* copies in bryophytes: one *RTFL* in *M. polymorpha* and two *RTFL*s in *P. patens.* Thus, studies on the RTFL family in bryophytes can increase our understanding of the function and evolution of RTFLs compared with other seed plants.

In this study, we identified subfunctions of motifs in the RTFL family. Future studies should explore the truncation or addition of specific motifs/motif patterns to characterize motif subfunctions. Meanwhile, the effects of representative orthologous RTFLs with similar motif patterns as the reported RTFLs should be examined based on overexpression. Details on the cell size and number, as well as anatomy in different root zones, should be confirmed in *ROT4 o/x* and *OsRTFL3 o/x*. In addition, plant hormones play an important role in the regulation of root growth. Studies on hormones such as auxin and cytokinins also reported abnormal root phenotypes with shorter or longer roots (Petricka et al. 2012). The phenotypes of cellular structure coupled with related candidate hormones should be examined to increase our understanding of root phenotypes in the above two RTFL overexpressors.

## Electronic supplementary material

Below is the link to the electronic supplementary material.

Supplementary material 1 (PDF 2510 kb)

Supplementary material 2 (DOCX 104 kb)

Supplementary material 3 (XLSX 71 kb)
