# Supplementary material for: Comparative analysis of the RTFL peptide family on the control of plant organogenesis
Source: J Plant Res. 2015 Feb 21;128(3):497–510. doi: 10.1007/s10265-015-0703-1 (PMC4408365; doi:10.1007/s10265-015-0703-1)
Supplement: Supplementary file 1 — Supplementary material 1 (PDF 2510 kb) [file 10265_2015_703_MOESM1_ESM.pdf]

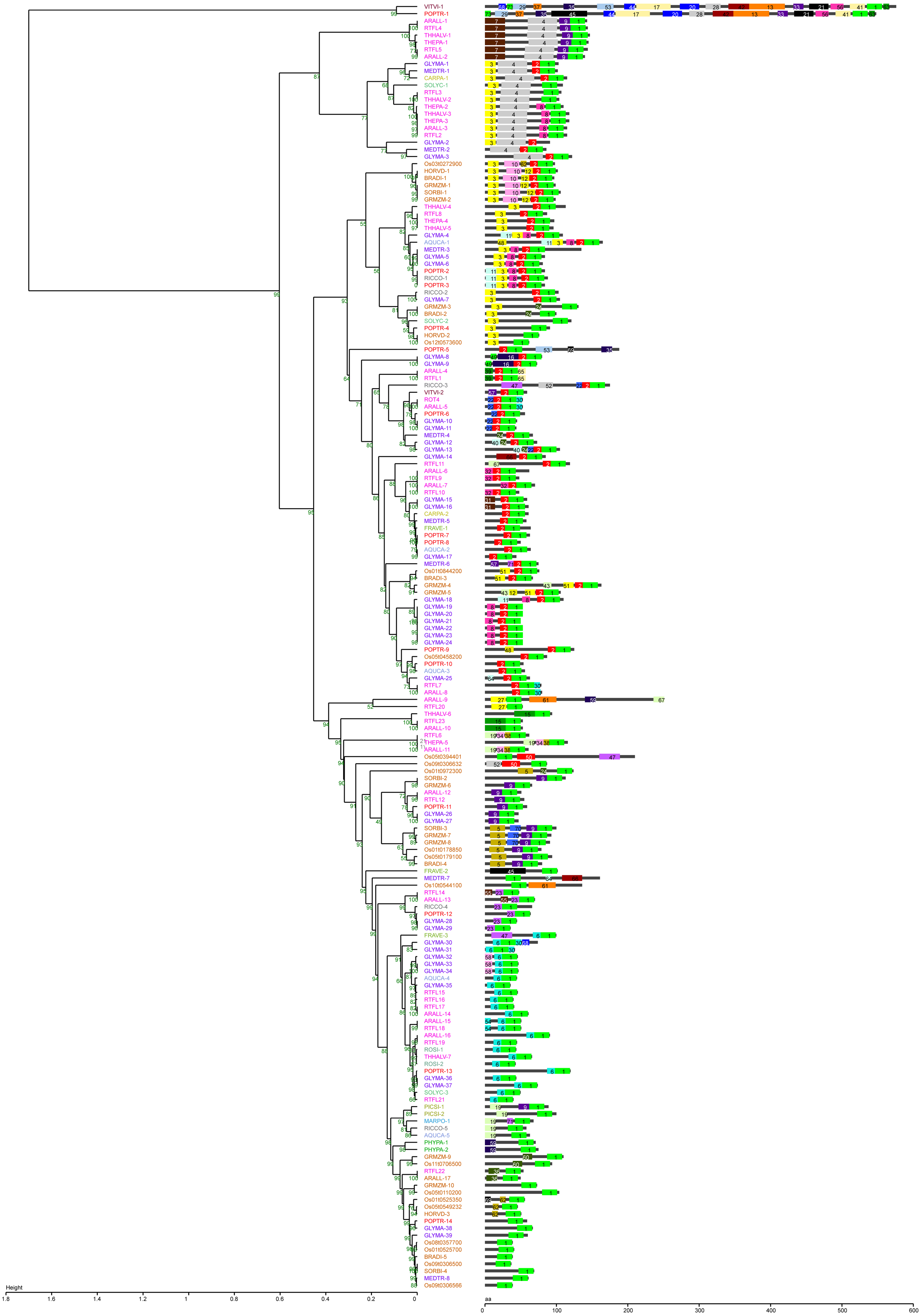

**Fig. S1** Comparative analysis of 180 RTFL members among land plants

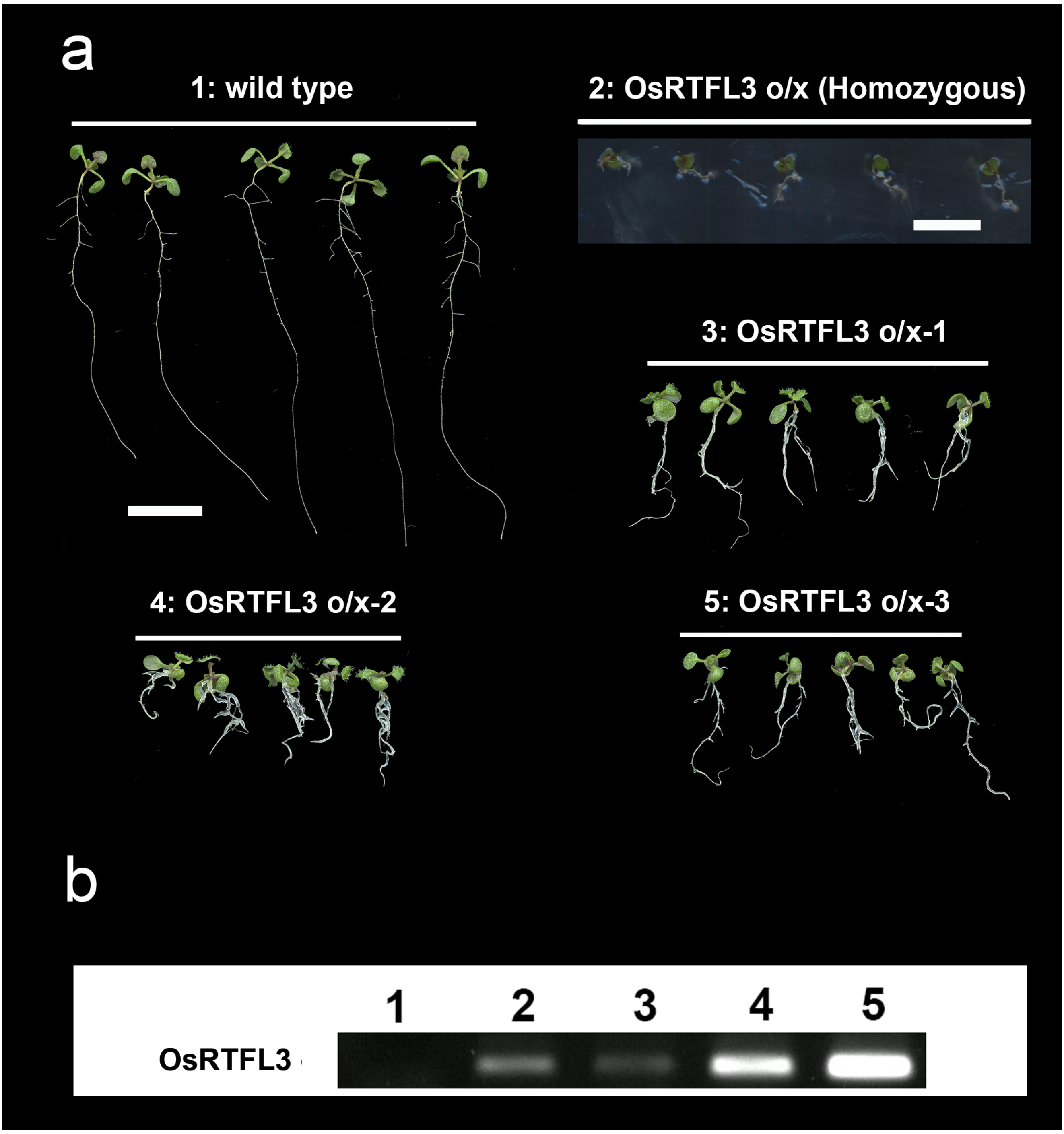


**Fig. S2** Root growth of wild-type and four independent *OsRTFL3 o/x* lines.
**a** Root phenotypes of 7-day-old plants. Only seedlings with strong phenotypes were selected from segregated siblings for *OsRTFL3 o/x*-1–3 (heterozygous lines). Scale bars; left (for panel 1, 3-5): 1 cm; right (for panel 2): 1 cm. **b** Presence of the *OsRTFL3* transgene in *OsRTFL3 o/x* lines. Results of genomic PCR of wild-type and *OsRTFL3 o/x* lines are shown. Lane 1: wild-type; 2: *OsRTFL3 o/x* (homozygous line); 3–5: *OsRTFL3 o/x* -1–3 (heterozygous lines). In each line, genomic DNA was isolated from a mixture of 10 individuals
