# Supplementary material for: Comparative analysis of the RTFL peptide family on the control of plant organogenesis
Source: J Plant Res. 2015 Feb 21;128(3):497–510. doi: 10.1007/s10265-015-0703-1 (PMC4408365; doi:10.1007/s10265-015-0703-1)
Supplement: Supplementary file 2 — Supplementary material 2 (DOCX 104 kb) [file 10265_2015_703_MOESM2_ESM.docx]

**Supplemental Table 1**

**Full-length amino acid sequences of 188 RTFL family members used in comparative analysis**

**MARPO**

- MARPO-1

MGQCYTAQQKPRERWIKKSGKGGPAGRYSQRESYGNGCFALVREQRARFYIIRRCITMLLCWHKYGGS

**PHYPA**

- PHYPA-1

MGQFYERDSGTSDAWKSTAKKRRSSGSFYSVRSNSITNVTKNTLAFIRHQRGKLYILRRCVIMLITVAREK

- PHYPA-2

MGQCYGKETLTSDENDSWKNTAGKRRSTGGIYSLHTNCISNGTKNTLTFIRHQHSKLYIVRRCITLLLLWHKYEK

**PICSI**

- PICSI-1

MSGAVKKMGQCFSGPAKTEGQWKEAKEEPGGSTEAEAYPRKRSKASSAPTDHGLGKRCA

- PICSI-2

MVLSERYWSTRSIVKKMGHCFSGHAKAERQWKPTKKEPRGPTPAAAYSRKRSAKRASVQ

**RTFL members of *Oryza sativa***

- Os03t0272900

MEFYVDDKWKFSKKSRNNGSRRIPGGSGAGGDPFLKRSASSRDQVIGRGRVGSGGGGGAAAAPSSFSSRCAGLVKEQRARFYIMRRCVTMLVCWKDCS

- Os01t0972300

MEDERWKLSSSKGRSKSGRSCSSSSNYYYHSSDFNSSNATTLSRSYSASVTASRHATTAWSAAGAGGGGASSSSSSQHQHQQQQQQSNNSQRLSKKCVEAVKEHRARFYIVRRCVSMLVCWRDY

- Os12t0573600

MEMCMDDKWKLTKKGSRRLEEGRASRGPSRSVPGRLASLVKEQRARFYIMRRCVTMLVCWRD

- Os01t0844200

MELYAVVKPCRLYKKRSSSGGGGKVAMCVRSGGDGGAGKSRPSFTCRCVRLVKEQRARFYIMRRCVTMLVCWHEYQ

- Os05t0458200

MEAMESRAEDQGVKRREPCKKRIGRTAGAGSEAGNGSRHQASCSPPPPPSSSFPRRCARLVKEQRARLYIVRRCITMLACWRDVDYL

- Os05t0179100

MELAAIASASASICSAYRHLSATPADDNGDDNGARPLSSSEELSKSSSSSRRMAKPAELRRRCYAVLKQQRTRLYILRRCVSMLLCWHEHDLSD

- Os05t0110200

MEAAAGGAREVHVMKKTCKNLKTKEDAAGGNVAVRKEQVMKTTKGLKKSPPPPSSSPEQVKMPCRSYSAENIKHRLTKTVKEHRARFYIIRRCIQMLICWRDEY

- Os01t0525350

LYIYREIERRRRRRGGEMKKQEKEGGGGGISKVVREHKARLYIIRRCVVMLLCWHD

- Os10t0544100

MRQCASASSSTSRPPEAAGEEGKRRRRRRGWLLQAAAREQRSRFYIFRRCVAMLLCWYKYRNITPYNEKANTGPDAAKRDRPPLDQQRDTDENTLRECQKRRLQEEKRRKTAPPSVGTQRSQDWASKTGLSSGNHP

- Os09t0306500

MEKVKKKRGKLQNVLREQKARLYIIRRCVVMLLCWSD

- Os01t0178850

MELSSSASSVCSAYRHLSSSSAMGPTPRRRRRGKVVGGGCAGVGLAGRCNAVLKEHKTRLYILGRCVSMLLCWHNHDSD

- Os09t0306566

MKTVNQGKQRGRMTRALKEHRARLYIIRRCIVMLLCWHE

- Os05t0549232

MVKLRIHGKNRAGGGGGGGLSRMLREQKARLYIIRRCVVMLLCYHD

- Os01t0525701

MKLGSQRRRGGFSKSLKEQRSRLYIISRCVVMLLRWHD

- Os08t0357700

MKVVSQVKQMGRLNKALKEKRAKLYIIRRCVVMLLRWSD

- Os01t0525700

MKLVGSERRQRGGGFGRAIKQQRARLYIIQRCVVMLLRWQD

- Os09t0306632

MAYPLLCHYIKAPHSSFPLIPHTSHYILQLVYLHLFHPLPCTQHSHQTMKIEGRRGQMGRLNRAFREKRARFYIFRRCVIMLLRWSD

- Os05t0394401

MEESKESSKLERLRSTAKQQKGKLYIIKICISMLICGSPKAALLTFIITQKIQTDRSHIEVFTQSGWQQFLNAQTTISYQCKSIGGERGTPHTGDRSGDRRARDGSHGDRPHRRERKGGGASGREVGEASTARSVSQRPDPCPGGRLGGCRRWSSSSSLKLGAGDPELLKAGGRGWRWPPPVGDGGRDTAAHLLLASARRRALLTAATHR

- Os11t0706500

MEEVESSYRSEMMDGEGWKMMQPNTNKQLVQIKTRSSSMQVVKEEIEEEEVAGGRGRRRGGLRRSVSGRVREQRARLYIMRRCVSLLISSTFHD

- Os01t0525800

MKLIGSGSGSGGQRRGIARALKEHKARLYIIRRCVVMLLRC

**BRADI**

- BRADI-1

MEFYVDEKWKFSKKSRNNGSRRVPGGGGGDPFLKRSASTRDQVIGRRAGAAGAASSAGGCAAPSFSSRCAGLVKEQRARFYIMRRCVTMLVCWKDCS

- BRADI-2

MEGDDKWKLSKKGRSRSGRNYFSSDAGGTSGTGGLSRSFSASVAGTRDPGGASSSKKEQQEQEQQGRRLSKKCVEAVKEHRARFYIVRRCVSMLVCWRDY

- BRADI-3

MELYAAEDKQPRKKSGKAAMSVRGNGNGNGKSRPSFPGRCARLVKEQRARFYIMRRCVTMLVCWRDA

- BRADI-4

MELAASASASVCSSYHHLSTQADADDGGAPSTPRRRKHAAAGCGLRRRCYTVLKQQRTRLYILRRCVSMLLCWNEHDLSD

- BRADI-5

MRVGAHDTKMKGLKRALKEQKARLYIIRRCVAMLLSWHD

**SORBI**

- SORBI-1

MEFYVDEKWKFSKKSRNNGSRRVPGAGGAGGGDSLLKRSSSMRDVPAIGRRGSGAAGAAAAAAAAGGCAPQPSFSSRCAGLVKEQRARFYIMRRCVTMLVCWKDCS

- SORBI-2

MPVRVRVRLARLAVSVCVARGGGAGRPSWCVVTARNACIMRMARVSSMESISASVPARSAMCCSTYGRRRRRQKAVGGFAGRCNAVLKQHKTRLYILGRCVSMLLCWHDHDAD

- SORBI-3

MELAASASTSVCSAAYHHLSAADAAAADRGGDSSSGAQGETTTTRLERRRRTKRAGGGGGGCAGLRRRCYAVLKQQRTRLYILRRCVTMLLCWHEHDLSD

- SORBI-4

MAGVDGHLILSAARRTVLNHGGRCSSAPIKGLLAAMEQQRRGKLQRVLREQKARLYIIRRCVVMLLCWSD

**GRMZM**

- GRMZM-1

MELYDDEKWKFSKKSRNNGSRRVPGAGAGGGGDSLLKRSSSMRDAPAIGRGAAAGAGGCAAQQPPSFSSRCAGLVKEQRARFYIMRRCVTMLVCWKDRS

- GRMZM-2

MELYVDEKWKFSKKSRNNGSRRVPGSGGGDSLLKRSSSMRDVAAIGRRGTGGAGAAAAGGCAPQPSFSSRCAGLVKEQRARFYIMRRCVTMLVCWKDCS

- GRMZM-3

MDSSSSAGAGDDRWRLSKKGRSRSGRPHAMDAAAGSVLSRSYSASVSSSRSTAAGTSTAAATGSSAAAPSSQQQQQQQQAGAAACPAESSSSSSSSSSRLSKKCVEAVKEHRARFYIVRRCVSMLVCWRDY

- GRMZM-4

MISARDSNNIQTKPQVASVWGWGRERLQPRTSSSSLHYITNRLCSYAMLCPSSTYKTNRRSTHATHSLYCLSPSSSPSEKGRHPSIHPSMELFAAAEEKPCRLFKKRSGGGGKGGMGVRADGSGKGGSFSGRCARLVKQQRARFYIMRRCVTMLVCWREREYA

- GRMZM-5

MRFLHIQDHRRSKHTSLSLTHSPHSTGHPSMELFAAAASAEDQKPCSSRLFSRKRSGGKAGMGVRGADKGRSSFSGRCARLVKEQRARFYIMRRCVTMLVCWREYA

- GRMZM-6

MARVSSVESISASVVTGSAVRSAYGRRQKPVGGLAGRCNAVLKEHKTRLYILGRCVSMLLCWHDHA

- GRMZM-7

MELAASASASVCSAYHHLSVPAAAADAAATDRGGAQETTTTRLDRRRGKRAGGGCAGLRRRCYKVLKQQRTRLYILRRCVTMLLCWHEHDLSD

- GRMZM-8

MELAASASASAVCSAYRQLSAPAAAADRGVGGDDAQETATRLDRRRRKRGGGCAGLRRRCYAVLKQQRTRLYILRRCVTMLLCWHQHDLSD

- GRMZM-9

MHRRGSSRCSSTSTSTSWDGDMKLLTRKKKEKPNTGEEGLGSRRKERENRVQLQAEAEAEEEEEDAAAPRRRLRRSVSMGSRLASAAREQRARLYIMRRCVSMLVRWKHD

- GRMZM-10

MEMHMDDKSKTPLSKKGSRRSASASAAAGLKSRPSRAAAGRSVPGRLAGLVKEQRARFYIMRRCVTMLVCWRD

**HORVD**

- HORVD-1

MEFYVDEKWKFSKKSRNNGSCRRVSGGGGGGGGGDPFLKRSASTRDQAIGRRGSASAAA

- HORVD-2

MEMFMDDKWKLSRKGSRRSAAVAPAAATGSPVGVKGRTSRGSGRSVPGRLASLAKQQRA

- HORVD-3

MNDVRKQEKQKGRGGGGGRSGGGRLARMLREHRARLYIIRRCVVMLLCHHD

**RICCO**

- RICCO-1

MASSLTHSASAPPQFYFDEKWKLSKKEGSSRSVRSSTSPLMKNSSSSSQRRCSFTRKCA

- RICCO-2

MDEKWKPSKKEGSSSFVRSFSTKSSSSKSPLLRSSSLKISSSPSSSSSSSNNNNKCPLP

- RICCO-3

MGPRRISNMRGEMAGQEHCFILCLHMLHHSLLTAGGLQWPQTIPNANTVERTQNPKISS

- RICCO-4

MASQRSAWIRFPKFRSWQRCSRLVKEQRTRLYIIWRCTVILLSWDDNHAKEQKNRAVAE

- RICCO-5

MGQCASGRRVVKVREDWDGGVSRGRHGWITIVKEQRSRLYIVRKCVVILVCWHKYGNS

- B9RYQ5_RICCO

MELSTSKRKKASPSPKRQTRVQQNSKGLKGTRARLYIIRRCITMLICWKEHRDD

- Q1KUS6_9ROSI

MESVRNSSEK KTSYSRRLGKYMREQKGRIYIIRSCVAMLLCWHD

- Q1KUW1_9ROSI

MERIRSSEEKKSYSRRLGKYLREQKGRIYIISRCVVMLLCWHD

**CARPA**

- CARPA-1

MDDKWKPSKKEASSSYHYSASSSSSSSSSSKSVFTRSFSTKSSSKSPLLIKSSSTKCSSSSSSSSSKCPLPRSYSQKGSSITRKCSSLAKEQKARFYIMRRCVAMLVCWHKHGDS

- CARPA-2

MADFQLRLKPATRRFNTPLKKQQQHGFTRKRASLIKEQRARLYVLRRCATMLLCLYINGDD

**GLYMA**

- GLYMA-1

MDEKGKVWKKEAGSSKSSLFSRSCSTRGSSSNSSSNSPLLIRSLSQKSSSTSSKTTNIPRSFSQKNPSIGKKCTSIAKEHRARFYIMRRCVAMLVCWHKHGDS

- GLYMA-2

MDEKRKASKKDTGSFSSPGSLFSRSTSTSNSPLLRSLSQKSSSSSSKCNNNNLPRSFSQKNPSIGRKCTKLAKEQKARFYIMRRCVAMLVC

- GLYMA-3

MSHSDFFLLSYHNLLSSSSSYTQRIRLSVRKSSSKKDTGSSSTRSLFSRSTSTSNSPLLRSLSQKSSSSSSKCNNNNLPRSFSQKNPSIGRKCTKLAKEQKARFYIMRRCVAMLVCWHKHGDS

- GLYMA-4

MVSSNCAPTCSNGTTTAGNNNNNNNNTDQTTAPPLYFSNSEKLRLPKKEGRSSTTSSPLIKNSSSSSSTQRKCAFARKCARLVKEQRARFYIMRRCVIMLICWHEYNDS

- GLYMA-5

MASSVSTTPPMYFDEKWKLSKKEGSTRSRSSSTSTPFIKNSSSSQRRCAFASKCAKLVKEQRARFYIMRRCVTMLICWRDYSDS

- GLYMA-6

MASSVSTTPPLYFDEKWKLSKKEGSTRSRSSSTPFIKNSSSQRRCAFASKCARLVKEQRARFYIMRRCVTMLICWRDYSDS

- GLYMA-7

MDEKWKVSKKEAGSSTSSSKSSLFSRSCSTRGSSSSSSSNSPLLIRSLSQKSSSTSKTTIPRSFSQKNPSIGKKCTSIAKEHKARFYIMRRCVAMLVCWHKHGDS

- GLYMA-8

MNTRHLICSYIINFRKNKTNHHKEYLFIERDMMQEDISSCTRHVFEPFKSFGRRCTRLAKEQRARFYIFRRCITMLVCWRE

- GLYMA-9

MLHIINFRKNKTNHHKKYLFIERDMMQEDISSCTRHVCEPFKSFGRRCTRLGKEQRARFYIFWRCITMLVCWRE

- GLYMA-10

MQQQHQVCDPCKSFGQKCSHLVKKQRAKFYILRRCVAMLLCWHEH

- GLYMA-11

MQQQHQNCDPCKSFGQKCSQLVKKQRAKFYILRRCVAMLLCWHEH

- GLYMA-12

TSTQLARKSFMAVKESNTSQNIQQQQQQQQEACDPCKSFGQKCSHIVKKQRAKFYILRRCIAMLLCWHERGDT

- GLYMA-13

MSTLLYKYLVEAVATMFSTTIFTALWCQIRVSTQLARKSFMAVKESNTSQNIQQQQQQQQQQEACDPCKSFGQKCSHIVKKQRAKFYILRRCIAMLLCWHERGDT

- GLYMA-14

MGHQIKKVASIDDLFEPFTYILIPTQSHKKGIKEGNEFLFFPRFLERNPSIGSKCTKLAKEQKARFYIMRRCVAMLVCWHKHGDS

- GLYMA-15

MAEFLNYNKCKQGTKVPAKRKGYGISSKCASLVKEQRARLYILRRCATMLLCWYIQGDD

- GLYMA-16

MAEFQNYNKCKQVTNKVVQAKRKGYGISSKCASLVKEQRARLYILRRCATMLLCWYIQGDD

- GLYMA-17

MKRKGHDDGFCNKCASLVKEQRARLYILRRCATMLLCWYIQGDHD

- GLYMA-18

MASSNCAISTCTSGTTAGINNNTDQSTVPPIMYLSNSEKWNKLSKKEGRSSTTSSSSLMKNSSSMSSSSSTQRKCAFARKCARLVKEQRARFYIMRRCVIMLICWHEYNDS

- GLYMA-19

MSQQTPSSSLMKNSSSSSTQRKCAFARKCARLVKEQRARFYIIRRCVIMLICWH

- GLYMA-20

MSQQTSSSSIMKNSSSSSTQRKCAFARKCARLVKEQRARFYIMRHCVIMLICWH

- GLYMA-21

MTSSSSIMKNSSSSSTQRKCAFARKCARLVKEQRARFYIMRHCVIMLICWH

- GLYMA-22

MSQQTSSSSIMKNSSSSSTQRKCAFARKCARLVKEQRARFYIMRHCVIMLICWH

- GLYMA-23

MSQQTSSSSLMKNSSSSSTQRKCAFARKCARLVKEQRARFYIMRRCVIMLICWH

- GLYMA-24

MSQQTSSSSLMKNSSSSSTQRKCAFARKCVRLVKEQRARFYIMRRCVIMLICWH

- GLYMA-25

MEEKGFHLFLHLVWVNSSSSSSTQRKCAFARKCARLVKEQRARFYIMRHCVIMLICWHEYNDS

- GLYMA-26

MTLSQSCRKTSLRKRCYLVAKQQKTRFYIFGRCIAMLLCWHDHTVSD

- GLYMA-27

MALSQSCRKTSLRKRCYLVAKQQKTRFYIFGRCIAMLLCWHDHAISD

- GLYMA-28

MAANVFSVRGSKVRSWERCSKQVRQQRTRLYIIWRCTVLLLCWHE

- GLYMA-29

SSKVRPWGRCSKYIRQQRTRLYIIWRCTVLLLCWHE

- GLYMA-30

MSKDFMRVSKKKVSCRRLGGNLKEQKGRLYIIRRCVVMLLYWHDYNHSHQQELFTFLFAAHHIMDDHTLESSKS

- GLYMA-31

KKVSCRRLEGYLKQQKGNLYKIRICVLMLLCWHDYNHSHEQP

- GLYMA-32

MKMGSPSMGGSKRRLSSRGLGGALREQRARLYIIRRCVVMLLCWHD

- GLYMA-33

MKMDSASMTGSKRRISSNRGIGGVLREQRARLYIIRRCVVMLLCWHD

- GLYMA-34

MKMDSASMTGSKRRISSNRGIGGVLREQRARLYIIRRCVVMLLCWHD

- GLYMA-35

SKRRLSSRGLGGALREQRARLYIIKRCLVMLLYWHD

- GLYMA-36

MSKDSTRGSKKKVSCRRLGGYLKEQKGRLYIIRRCVVMLLCWHD

- GLYMA-37

MGFQVSTHTLSLYTTKCKLLTKKGRSIDSKMSKDSMRGSKKKLLCRRLGGYLKEQKGRLYIIRRCVVMLLCWHD

- GLYMA-38

MFEKEQRGNEQSPGFIIHAEKMKIETETIRVSKRKLMGKGMGALLKEGRGRFYILRRCIIMLLCSHD

- GLYMA-39

MGQSASRTTHNRGEFSGGGCPVHSKRQGRFAIIKEHKSRLYIVRRCILMLICWHKYKISE

- Glyma15g32260_1

KKDFTSSFTLCGSSNSSSSSTQRKCAFARKCAWLVKEQRARFYIMRRCVIMLIC

**MEDTR**

- MEDTR-1

MDEKWKLSKKEKSSNTSSTKSSLFSRSCSTRGSSSNSPLLLKSFSQKSSSTSNNSGNLSRSYSQKNPSIGKKCTNIAKEQKARFYIMRRCVAMLVCWHKHGD

- MEDTR-2

MDEKRKTSSKSLFSRSSSTRSSTSNSSLLRSSSSSSKCNLQRSFSQKNPSSIGRKCTNLAKEQKARFYIMRRCVAMLVCWHKHGDN

- MEDTR-3

MASSSSMESATPQPQPPLYFDEKWKLSKKEGSTRSKSSSSSFIKNTSTTQRKCAFARKCARLVKEQRARFYIMRRCVTMLICWRDYRTREGRGCSHHNADSDAGGLSLMVGSGTDDELLTMAPQHRAKYSVKRKK

- MEDTR-4

MAMAAEKINNTNQITETNHQQQEEQTCFPCKTFVQKCDHFVKNQRAKFYIFRRCIMLLLCWNESSSD

- MEDTR-5

MAEFQNLKYKQGEKVATKKKRNGISSKCASLVKEQRARLYIVRRCATMLLCWYIQGED

- MEDTR-6

MQLLEKATNFIRVQKNQQNQPQGATFNEMKNKESHVNGPFNSFGQRCSRKVRELRVRFYIIRKCVRMLVCWKDIE

- MEDTR-7

MDNSYSKTYPSNCEELANSSLKKRFLKRAKEERSRLYILKRCIIMLLCWHNVKEGKEKDTVSHEDRDTSWLPSTGPFFWSSETKRGFHYFFCLGLPYFIHKFISSQPSPFSLTLFTSPSAIYDINHLVLWLFDSNFMFNFSVAGNVCCTIYYVGQVQVQTN

- MEDTR-8

MGYTQVESFKLKKTSNPMGRSKLKRSSGLSSNKGFGGVLREQRARLYIIRRCVIMLLCWHE

**POPTR**

- POPTR-1

MKVPAPSPQNSSSSNNQSQNPKPVSCNNTNNNSRNISFNPSNRNLCYEPTSVLDLRRSPSPARAGKPASATDPIEWEDHVLQTLDWDSIMRELDFHDDSAPALIKNFPQFGPCSEPQIQSHNLPEFTASQQIDATQFLNSEFNDMYINSIPTHNLTSLDLSHSFHNNIGNWNAGSDFIQELIKAADCFDSNELQVAQVILERLNHRHQSPNGKPLQRAAFFFKEALQSLLTTGSTRPQTNPVVPSWSNTVQTIKAYKAFFSISPIPMFTDFTTNQAILDSLNGNSVFLHVIDFDIGFGCHYASLMRELVDKADSCNKITTPLLRITAVVTEDTVIGTKLIKERLSQFAHELKIRFHVEFVLFPTFEMLSFKAIKFFEGEKIAVLLSPTIFRHLGSTNNVTMFVNDFRRVSPSVVIFVDSEGWTESGARLSFRRNFVNCLEFYSMMFESLDAAVITAGGDWARKIEMCLLKPKILAAVEGCGRRMVSPWREVFAGAGMRAVQLSQFADFQAECLLGKVQVRGFYVAKRQAELVLCWHDRPLIATSAWKC

- POPTR-2

MTNTLAHSASAPPQLYFDEKWKLTKKEGSSRSRSSTSSLMKNSSQRRCSFTRKCARLVKEQRARFYIMRRCVTMLICWRDYNDA

- POPTR-3

MAITLTHSASEPPQFYFDEKWKLSKKEGSSRSRSSTSSLMKDSSQRRCSFTRKCARLVKEQRARFYIVRRCVTMLICWRDYSDA

- POPTR-4

MDEKWKLSKKEGSSSFTRSFSTKSSSSKAPLLRTSSLKSSSPKCPLPRSFSQKNSSISHKCSSLAKEQKARFYIMRRCVAMLVCWHKHGDS

- POPTR-5

MAEVKLLQKNITESPAKKKRHGFTRKCASLVQEQRARIYVLRRCATMLLCCCCDLKVFESCHFSSKVKLLRPFALCLVPGVSYENVTWMLFFEFSYSAPVLSTHSVAGISRFDFSNWYIWFEIYNAPLENDTIRSWHIIGRLGGCNSMNMQGANVTPATFYNSGDFEIQDHIPITLNLESLYLSIIYS

- POPTR-6

MAGKEKSETQLHGCEPCRSFGQKCSHLVKKQRGKFYIVRRCIAMLICWHERERGEP

- POPTR-7

MAEIKLQVYNKSSGAKKAPATRRSKGHGFTNKCAALVKEQRARIYILRRCATMLLCWYIQGDD

- POPTR-8

TGAKKAPATRRSKHSFTNKCAALVKEQRARIYILRRCATMLLCWYIQGDD

- POPTR-9

MTVTSGFARTESQKQRSPTSMQIERTPGFTTSDDKHFNYYTDRALSFVEQWAQLSSLNQLMTAPLLRSSSLKISSPKCPLPRSCSQKSSSISRKCSSLAKEQKARFYIMRRCVAMLVCWHKHGDS

- POPTR-10

MKQENTGFCSKHVRDPCRSFGRRCSSLVKEQRARFYILRRCVTMLVCWHDYGEP

- POPTR-11

MRALQVQTTTSTAQETKIQMAADALSMRSMKLRSWQRCSKQIREQRTRLYIIWRCTVMLLCWHD

- POPTR-12

MVAGSLVVCGGLGACSLELWTTLHTLLRSCLSSSSPSITHSLQALLLLVLKCILLLSEKAKKLSASTSQKTKTMKMTSQATMEESKKKMSCRRLGGYLRQQKGRLYIIRRCVVMLLCWHD

- POPTR-13

MVMKPENITSFGDQLRVRDSCRSLGERCCRVVKELRARFYILRRCVAMLLCSNEVNEET

**VITVI**

- VITVI-1

MCAGSGRVECDLSIGSLPLLFFFLFLAFPLAMKVPVPAQNNHSPNPKPLSCNNTTRNTAFRSPATDTINVCYEPTSVLDLRRSPSPVADKAATFPGITAVSDVSLPLSEEPGFQWEDHGMHNLDDWDPTVWDFVLRDDSAPVFGSVPQLGPCDPQFPHLPLPDLPPSQPIYHTQLLPFDFTLSEISSNQNHNFNLNSFHWNVGSEFVEELIRAAECFGSNNSQLAQAILARLNQRLRAPVGKPLQRAAFYFKEALHSLLTGSNRKSHSSASEIVQTIKAYKAFSMISPIAMFSNFTASQALLEAVDGSLFIHIIDFDIGLGGQYASFMKEIADRSEACKVNPPVLRITAVVPEEYAVESRLIKENLFQFAQELKIEFRIEFVLIPTFEVLSFKAVKFIDGEKTAVNISPAIFRRLGTTNNIAGFFCDLRRISPQVVVFVDGEGWTDSGATSFNRNFINGLEFYTAMLESLDAGGAGAGGDWVRKIEMSLIQPKIFAAVGDVGRRVTAWRELFSGAGLGQVQWSQFAESQAECLLGKSQVRGFHVAKRQAEMLLCWHGKPLVATSAWRWFACNSILV

- VITVI-2

MAAKENNIGLQSHQQDRHPCRCKSFGQKCSHLVKKQRAKFYILRRCIAMLVCWHERGDP

**FRAVE**

- FRAVE-1

MDVAHVQHCNKSTKRSRRGFSGKCAALVKEQRARIYILRRCATMLLCWTPVSAGLILMCGLSST

- FRAVE-2

MQGLRTEGCFGILDFGKFDAHGFENPDEDEFGWVGARIQLEISRIVEALMGMAAGSSSSSSNDKKRSKVSSRKLGGYLRQQKGRLYIIRRCVVMLLCWHD

- FRAVE-3

MTPPKGQPEANICQPPPHIMFSPLSPHTSPSPLSLNISNNPVGIVFDSMATAGNEIPTRRSRSSRRSTSTLKVGSLGRCLKEQRGRLYIMWRCTVMLLCWDD

**RTFL members of Arabidopsis**

- ROT4

MAPEENGTCEPCKTFGQKCSHVVKKQRAKFYILRRCIAMLVCWHDQNHDRKDS

- RTFL1

MGEENSTSGTCKPSKTFKAKCSHMVRKQRAKFYILGRCLAMLVCGRGRDRERDRILI

- RTFL2

MEEKWKLSKKDTTASSSSSKSKFSRSFSTSASSTKSPIFVRSSSTKCSVPSSSSSSSSSSSISRSFSRKERRSSSSSSSSITQKYSSLAKEQKARFYIMRRCVAMLVCWHKHGDS

- RTFL3

MDEKWRLSKKDALAASCSSSSTSSKSKFSRSFSTSASSSKAPAFVRSSSTKCSVPSSSSSSISRSSSKKEKGSITQKYSSLAKEQKGRFYIMRRCVAMLVCWHKHDS

- RTFL4

MDVEKLWNHTKKDSIFQTTHFSSSSKPFFTRSFSTKTSSSPSSKSHFTRSFSTKPSSSSSSSDLIFRRSFSAKPKTSKSLLLSRSCSTKSSADLSSKSSSLSRILSKKGASVTGKCFKVAKEHKSRFYIIKRCVLMLVCWHKHS

- RTFL5

MDDENLWKVVKKDSIFETTHFSSKPVFTRSFSTKTSSSSSKPVFTRSFSTKPTSYSSSEPIFRRSFSAKPTSSKSPFLSRSGSTKCPVDTSSTSKCSISRSLSQKGASVTRKCRNMAKEHKSRFYIMKRCVLMLVCWHKHACDS

- RTFL6

MGQCSSATKMRRKRKREEECCRESMERRNKGCLAMVKERRSRFYIARRCILMLLCWHKYANS

- RTFL7

MSRLRNSAQLQLSKKESLGDNGGALNTTRSSRQKQGKYGFTRKCGRLVKEQRARFYIMRRCVVMLICWTDHNNNNSDHS

- RTFL8

MASSSSLTRSGSVHLDEKWKLSKKDGGASRITRSSSTSSSSFNGKKQGRCAFTRKCARLVKEQRARFYIMRRCVIMLICWRDNYSDS

- RTFL9

MAEFKSKLNKGHAFTSKCASLVKEQRARLYILRRCATMLCCWYIQGDE

- RTFL10

MAGLKRKFNKGHAFTSKCVSLVKEQRARLYILRRCATMLCCWYIHGDE

- RTFL11

MCIILHSCTARLPQAQNLQIELKQDQVQASFKSNEKKNSIFIKLVSEKPMLINRRVESSNLLHSNMGGFLAKKTNSNSKIRNSFTSKCTSLMKQQHARLCIIRLCATMLLRSYTDHDDY

- RTFL12

MCLFMSNSSLPTKPNRKTRFGDRCLLMAKQQRTRLYILRRCVSMLLCWHDHSISD

- RTFL13

MSERRVGSYRKSTLRCWDWCKEQRTRAYIIWRCLIFLLRWDDY

- RTFL14

MAGTVVLRCCTSVTKVRTWKRCSKQIKEQRARLYIIWKCAVFLLSSHD

- RTFL15

MKTTGSSVGGTKRKMWSRGVGGVVREQKAKLYIIRRCVVMLLCWHD

- RTFL16

MGVLKRRVSSSRGLGGVLREQRAKLYIIKRCVVMLLCWQD

- RTFL17

MKMGGSKRRVSSKGLGAVLKEQRAKLYIIRRCVVMLLCWHD

- RTFL18

MEMKRVMMSSAERSKEKKRSISRRLGKYMKEQKGRIYIIRRCMVMLLCSHD

- RTFL19

MESIMSLKRKEKKSQSRRLGKYLKEQKGRIYIIRRCVMMLLCSHD

- RTFL20

MREKYTKEEAVKNWEKKKNKPSSPKGVGEFLKKKKGRFYIIGKCITMLLCSHK

- RTFL21

MKGTKKKTPCNKKLGGYLKEQKGRLYIIRRCVVMLICWHD

- RTFL22

MIVKLMGSNKDLHRQAKNNNNKLTPNRSLKETRSRLYIIRRCLVMLLCWREPRD

- RTFL23

MLVSNISGKLMSQLMEKMKERLEKMKRTVRQQRAKLHIIRICITMLLSSDDYS

**ARALL**

- ARALL-1

MDVEKLWNHTKKDAIFQTTHFSSSSKPFFTRSFSTKTSSSPSSKSHFTRSFSTKPSSSS

- ARALL-2

MDDEKLWKVSKKDSIFETTHFSSKPVFTRSFSTKPTSSSKPVFIRSFSTKPTSYSSSEPIFRRSFSAKPAPSKSLFLSRSSSTKCQADTSSASKCSISRSLSQKGASVTRKCRNMAKEHKSRFYIMKRCVLMLVCWHKHA

- ARALL-3

MEEKWKLSKKETTASSSSSKSKFSRSFSTSASSTKSPIFVRSSSTKCSVPSSSSSSSSSSSISRSFSRKERRSSSSSSSSITQKYSSLAKEQKARFYIMRRCVAMLVCWHKHGDS

- ARALL-4

MGEENSTSGTCKPSKTFKAKCSHMVKKQRAKFYILGRCIAMLVCGRGRDRERDRILI

- ARALL-5

MAPEEKGTCEPCKTFGQKCSHVVKKQRAKFYIVRRCIAMLVCWHDHNHDRKDS

- ARALL-6

MAEFKSKLNKGHAFTSKCASLVKEQRARLYILRRCATMLCCWYIQGDDSMDIEALSFGC

- ARALL-7

MDQQLCLPRFLHLNKETLIFDPMAGLKRKFNKGHAFTSKCVSLVKEQRARLYILRRCATMLCCWYIHGDE

- ARALL-8

MSRPRSSPQFQYLPKKERFGDDGGASRNTLSSRQKQGKYGFTRKCGRLVKEQRARFYIMRRCVVMLICWTDHNNNNSEDS

- ARALL-9

MKEKSKEEAVKNWDKKKNKPSSPKGVGEFLKKKKGRFYIIGKCITIFLSFNNINGKFQPKVQDDQHGASRCNVINQAAADTKEWLDNTINAAPSIGNHCNRRGRSDRWRKPPRGWIKCNYDVSGLGWIIRNDSGTFLDCGMGQFEGRVTTEEAECTALLWALGYRVVEFEGDNQNVVRIINNKEANPRLQHFLDTIWSWSKRFTAISFLFTHREQNSCADLLARNIVSNSNPWLLYHSCPHFLTQFVNNDI

- ARALL-10

MLVSNISGKLMSQLMEKMKERLEKMRRTVRQQRAKLHIIRICITMLLSSDDNS

- ARALL-11

MGQCSSTTKMRRKRKREEEGCRESMERNKGCLAMVKERRSRFYIARRCILMLLCWHKYA

- ARALL-12

MVKASCLSFTLRNMFLDIARGSMAGTVVLRCCSSVNKVRTWKRCSKQIKEQRARLYIIW

- ARALL-13

MIRPLHLHLQRKKKLIDISKMKMGGSKRRVSSKGLGAVLKEQRAKLYIIRRCVVMLLCWHD

- ARALL-14

MEMKKVMMSSSGRSKEKKRSISRRLGKYMKEQKGRIYIIRRCVVMLLCSHD

- ARALL-15

MPTIISPYKNSFEQVSSQSNNSSSKSLICYTSIVISTGFLWTQLDMESIIMSLRRKEKKSQSRRLGKYLKEQKGRIYIIRRCVMMLLCSCD

- ARALL-16

MKLMGSNKDLHKQVKNINKLTPNRSLKETRSRLYIIRRCLVMLLCWREPRD

**THHALV**

- THHALV-1

MDDQKLWKVSKKDSIFETAHFSSKPVVFTRSFSTKNSSSSSSSKPIFTRSFSTKHTSYSSSEPIFRRSFSAKPTPSKSPFLSRSGSTTTKCPNDASSSSSKCSISRSLSQKGASVTRKCRTKAKEHKSRFYIMKRCVLMLVCWHKHA

- THHALV-2

MDEKWKLSKKDALAASCSSSSSSKSKFSRSFSTSASSSKAPVFVRSSSTKCSVPSSSSSISRSSSKKEKGSTSSFSNLAKEQKGRFYIMRRCVAMLVCWHKHDS

- THHALV-3

MDEKWKLSKKETTASSSYSSKSMFSRSFSTSASSTKSPIFVRSSSTKCSIPSSTYSSSSSSSVSRSFSRKERRSSSSSSSSSSITQKYSSLAKEQKARFYIMRRCVAMLVCWHKHGDS

- THHALV-4

MKQQPTTRIFRGKIKNLRILRKMTRSGNSSSQFQLSERWKLSSKKESFGGEEGASKTTRSSRQKKQGKNGFTRKCGRLVREQRARFYIMRRCVVMLICWTDNHNNNDNSYSDHS

- THHALV-5

MASSGLTRSGSAPQFHLDEKWKLTKKDGFYGGASRITRSSSTSSFSISGGKKTQKQGRCAFTRKCARLVKEQRARFYIMRRCVIMLICWRDNYSDS

- THHALV-6

YLLHVGFRQSFMVFGHFVYMLEVELKCIGVCMYINVCVCMYICGCPTYLELMRQLMETMKERLEKMKRTMRQQRAKLHIIRICITLLISSDNNS

- THHALV-7

RKQFSHFFSTGKICNIEEIEMERIVMSSEKEKKRSLSRRLGKYMKEQKGRIYIIRRCVVMLLCWHD

**THEPA**

- THEPA-1

MDDEKLWRVSKKDSIFETTHFSAKPDVFRRSFSTKTSSSSSSSLSKPIFTRSFSTKPTSYPSSEPIFRRSFSAKPTPSKSPFLSRSGSTKCQADTSSTKCSISRSLSQKGASVTRKCRNVAKEHKSRFYIMKRCVSMLLCWHKHA

- THEPA-2

MDEKWKLSKKDASAASCSSSSSSKSKFSRSFSTSASSSKAPAFVRSSSTKCSVSSSSSSISRSSSKKEKGSSSSSSITQKYSSLAKEQKGRFYIMRRCVAMLVCWHKHDS

- THEPA-3

MEEKWKLSKKETTASSSSSSKSKFSRSFSTSASSAKSPLFIRSSSTKCSVPSSSSSSSSSSSISRSFSRKERRSSSSSSSSSSITQKYSSLAKEQKARFYIMRRCVAMLVCWHKHGDS

- THEPA-4

MASSGLTRSGSAPQFHLDDKWKLSKKDGFSGGASRITRSSSTTSSFSINGGMKTQRQGRCAFTRKCARLVKEQRARFYIMRRCVIMLICWRDNYSDS

- THEPA-5

MTVRCEKGRVITPSDPITIREGHVGKSSLQRIENRKEEDIKSVKKPSKRGRTTNMGQCSSTTKMRRKRKREKEEGCRESMERNRGCLAMVKERRSRFYIARRCILMLLCWHKYANS

**ROSI**

- RISO-1

MESVRNSSEK KTSYSRRLGK YMREQKGRIY IIRSCVAMLL CWHD

- RISO-2

MERIRSSEEKKSYSRRLGKYLREQKGRIYIISRCVVMLLCWHD

**SOLYC**

- SOLYC-1

MEYYMDEKWKLSKEDPNNNSYNSSSKSSLRRSYSQHCPISSDPPLPRSYSQKTSSSSKSRLSKSSSQRSSNFKSKCSTMAKEQKAKFYIVKRCIAMLVRWNKHDKHGDS

- SOLYC-2

MEYYMDERWKLSKDDPCCNSYSSSNNKSSSLFRSFSQKSPNSNISSLPRSLSQKNPHKSSLSRSSSQKSSTSKCRLTKSASQRCANFRSKCSNLAKEQKSKFYIVKRCIGMLVRWKKHGDS

- SOLYC-3

MKMNNVGGSTHDENSKKKMSSRRLGKFLKEQRGRLYIMRRCVVMLLCWHD

**AQUCA**

- AQUCA-1

MAGDKQKLKLRKTRNPKLFINTTDPHLHYHIFNLHRHSHHQSTTTTTTNPNFSHTVTKVRVLILYLMAIDSLSHSSSTHHGTSSLPPPPPPQFYMDEKWKLSKKECSRSRSSSTTSATPFMRNSSKRRCAFTRKCATLVKEQRARFYIMRRCVTMLICWRDYGDS

- AQUCA-2

MAAEQNMYNYSADRHVQYLESKTPGRNNGFSRRCASLVKEQRARLYILRRCATMLLCWYIQGDE

- AQUCA-3

MSMNEDSTSFCCRNVRDPCRSFAQRCSRLVKEQRSKFYILRRCVIMLVCWHECGET

- AQUCA-4

MKTSNAAMRSSKRRLSRGLGGVLREQRGRLYIIRRCVVMLLCWRE

- AQUCA-5

MGQCTSTRSKESKRWSDVSSTTSSSSSSEKRGWLALVRERRSRFYIVRRCVVMLLCWHKYGKY

- Aquca_092_00004

MAMLLFSRRNPKLGKWRQWSKYTQQQRARLYIIWRCTVILVCWQE
